# A new polysaccharide platform constructs self-adjuvant nanovaccines to enhance immune responses

**DOI:** 10.1186/s12951-022-01533-3

**Published:** 2022-07-14

**Authors:** Sisi Chen, Liu Yang, Xia Ou, Jin-Yu Li, Cheng-Ting Zi, Hao Wang, Jiang-Miao Hu, Ye Liu

**Affiliations:** 1 Institute of Medical Biology, Chinese Academy of Medical Sciences and Peking Union Medical College, Kunming, 650000 Yunnan China; 2 State Key Laboratory of Phytochemistry and Plant Resources in West China, and Yunnan Key Laboratory of Natural Medicinal Chemistry, Kunming Institute of Botany, Chinese Academy of Sciences, Kunming, 650201 Yunnan China; 3 School of Medicine, Kunming University of Science and Technology, Kunming, 650201 Yunnan China; 4 College of Science, Yunnan Agricultural University, Kunming, 650201 Yunnan China; 5 CAS Key Laboratory for Biomedical Effects of Nanomaterials and Nanosafety, National Center for Nanoscience and Technology, CAS Center for Excellence in Nanoscience, Beijing, 100190 China

**Keywords:** Polysaccharide, Self-adjuvant, Nanovaccine, SARS-CoV-2, HIV

## Abstract

**Background:**

Nanovaccines have shown the promising potential in controlling and eradicating the threat of infectious diseases worldwide. There has been a great need in developing a versatile strategy to conveniently construct diverse types of nanovaccines and induce potent immune responses. To that end, it is critical for obtaining a potent self-adjuvant platform to assemble with different types of antigens into nanovaccines.

**Results:**

In this study, we identified a new natural polysaccharide from the rhizomes of *Bletilla striata* (PRBS), and used this polysaccharide as a platform to construct diverse types of nanovaccines with potent self-adjuvant property. In the construction process of SARS-CoV-2 nanovaccine, PRBS molecules and RBD protein antigens were assembled into ~ 300 nm nanoparticles by hydrogen bond. For HIV nanovaccine, hydrophobic effect dominantly drove the co-assembly between PRBS molecules and Env expression plasmid into ~ 350 nm nanospheres. Importantly, PRBS can potently activate the behaviors and functions of multiple immune cells such as macrophages, B cells and dendritic cells. Depending on PRBS-mediated immune activation, these self-adjuvant nanovaccines can elicit significantly stronger antigen-specific antibody and cellular responses in vivo, in comparison with their corresponding traditional vaccine forms. Moreover, we also revealed the construction models of PRBS-based nanovaccines by analyzing multiple assembly parameters such as bond energy, bond length and interaction sites.

**Conclusions:**

PRBS, a newly-identified natural polysaccharide which can co-assemble with different types of antigens and activate multiple critical immune cells, has presented a great potential as a versatile platform to develop potent self-adjuvant nanovaccines.

**Supplementary Information:**

The online version contains supplementary material available at 10.1186/s12951-022-01533-3.

## Background

Traditional vaccines are constructed by pathogen culture (inactivation and attenuated vaccine), protein expression (protein subunit vaccine), nucleic acid amplification (DNA and mRNA vaccine) or chemical synthesis (peptide vaccine) [1–3]. These traditional strategies can only prepare separated antigens, but fail to assemble multiple separated antigens into an integrated architecture to improve the efficacy of vaccines in vivo. Even in a few cases, researchers realized the co-assembly among separated antigens, there still is lack of a versatile platform to conveniently assemble diverse types of antigens together and construct powerful vaccines with self-adjuvant property [4–6].

In our hypothesis, such a versatile platform for the construction of self-adjuvant nanovaccines should possess two critical functions. One is that there are abundant active groups on the surface of the platform to provide assembly sites between the platform and antigens. Another is that the platform as an adjuvant possesses the immunostimulation capability to enhance antigen-induced immune responses. To satisfy aforementioned requirements, we selected natural polysaccharides as a potential candidate platform. Firstly, amounts of active groups and the flexible molecular structure enable natural polysaccharides to assemble with other bioactive molecules such as peptides, proteins and nucleic acid [7, 8]. Secondly, some natural polysaccharides have shown their immunostimulating effects on macrophages, T lymphocytes and B lymphocytes [9, 10]. Moreover, natural polysaccharides universally distribute in various living systems and participate in the construction of cells and tissues, and have the satisfactory water solubility and safety. Some of them had even been used as pharmaceutical materials to manufacture medicines such as heparin, chondroitin sulfate and Ganoderma lucidum polysaccharide [11–13]. Together, we think that selecting natural polysaccharide as a versatile platform to construct diverse types of self-adjuvant nanovaccines is feasible.

In the current study, we identified a new natural polysaccharide (PRBS) from the rhizomes of *Bletilla striata*. By nuclear magnetic resonance (NMR) and FTIR, we revealed the chemical structure of PRBS, which possessed a long chain with molecular weight of 42.4 kDa and mainly consisted of 1,4-linkage *β*-D-mannose and *β*-D-glucose. We used PRBS as a versatile platform to construct both polysaccharide-protein nanovaccine against SARS-CoV-2 and polysaccharide-DNA nanovaccine against HIV. We demonstrated that hydrogen bond and hydrophobic effect determined the assembly of polysaccharide-protein and polysaccharide-DNA nanovaccine by isothermal titration calorimetry (ITC) assay and theoretical calculation. Moreover, relying on the potent immune stimulation capacity of PRBS, these nanovaccines can significantly enhance both antibody and cellular immune responses in vivo without the addition of adjuvants. Together, this study experimentally proved a proof-of-concept of using a natural polysaccharide as a versatile platform to formulate self-adjuvant nanovaccines.

## Materials and methods

### Reagents

The dried rhizomes of Bletilla striata were harvested in Pu′er city, Yunnan province, China. The voucher specimen was identified by Professor Zhi-Kun Wu in Kunming Institute of Botany, Chinese Academy of Sciences. Diethylaminoethanol (DEAE) Sepharose fast flow and high-resolution sephacryl S-200 were from GE Healthcare (Sweden). Monosaccharide standards (glucose [Glc], glucuronic acid [GlcA], mannose [Man], rhamnose [Rha], galactose [Gal], galactose acid [GalA], fucose [Fuc], xylose [Xyl] and araose [Ara]), 1-phenyl-3-methyl-5-pyrazolone (PMP), trifluoroacetic acids (TFA) and dimethyl sulfoxide (DMSO) were from Innochem Co. Ltd (Beijing, China). Lipopolysaccharide (LPS), T-series dextran and 3-(4,5-dimethylthiazol-2-yl)-5(3-carboxymethoxyphenyl)-2-(4-sulfopheny)-2H tetrazolim (MTS) were from Sigma-Aldrich (USA). The NO-detecting kit was from Beyotime Institute of Biotechnology Co. Ltd (Shanghai, China). Mouse tumor necrosis factor-α (TNF-α), mouse interleukin-6 (IL-6) and mouse interleukin-1β (IL-1β enzyme-linked immunosorbent assay (ELISA) kit was from Boster Biology Engineering Institute (Wuhan, China).

### Extraction and purification of polysaccharide

3 kg dried rhizomes of planted Bletilla striata were powdered to irregular particles and defatted with 85% ethyl alcohol (EtOH) three times, and were extracted three times by distilled water 1:15 (w/v) at 90 ℃ for 3 h per each time. All of extracts were concentrated with a rotary evaporator at 50 ℃ under reduced pressure and precipitated by adding 95% ethanol to a final concentration of 70% overnight at room temperature. Proteins in the extracts were removed by Sevage method. These protein-free extracts were lyophilized to obtain crude polysaccharide (250 g). A portion of crude polysaccharide (200 mg) which was then dissolved in 10 mg/mL of distilled water. After a centrifugation, the supernatant was harvested and injected into a 2.5 × 60 cm column of DEAE sepharose fast flow equilibrated with distilled water. The column came through a linear gradient elution with 0, 0.05, 0.3 and 0.5 M NaCl solution at 0.5 mL/min flow rate. Eluent (4 mL/tube) was collected by automatic collector and carbohydrates were monitored by high-performance liquid chromatographic—evaporative light scattering detection (HPLC-ELSD) assay. The eluate was concentrated, dialyzed and lyophilized to obtain sub-fractions of polysaccharide (100 mg). 0.05 M NaCl eluates were further purified by gel-permeation chromatography on a column of Sephacryl S-200 (2.5 × 100 cm) gel respectively. The eluent solvent was water with a flow rate of 0.25 mL/min. PRBS (60 mg) was purified, concentrated, dialyzed, lyophilized and stored at – 20 ℃ for further analysis.

### Determination of homogeneity and relative molecular weight

The homogeneity and average molecular weight of polysaccharide were determined by high performance gel permeation chromatography (HPGPC) in an Agilent 1260 HPLC system (Agilent Co. USA), which was equipped with evaporative light scattering detectors (Alltech ELSD 2000ES, USA) and TSK-GEL G4000 PWxL column (7.8 mm × 300 mm). Briefly, 1 mg/mL polysaccharide was eluted with water at 0.8 mL/min flow rate. The column was maintained at 30 ℃. The eluent was detected by an Alltech ELSD 6000 detector. Column calibration was performed with standard T-series dextrans (Mw: 5000, 12,000, 50,000, 150,000, 410,000 and 670,000 Da). The molecular weight was estimated based on the HPLC calibration curve from T-series dextran standards.

### Assay for monosaccharide composition

Monosaccharide composition of polysaccharide was analyzed by reverse-phase HPLC according to 1-phenyl-3-methyl-5-pyrazolone (PMP) derivatization method. 2 mg/mL polysaccharide solution was incubated with 1 mL TFA (4 M) at 120 ℃ for 4 h. These products were dissolved in methanol and evaporated to remove residual TFA, which was repeated four times. 50 μL polysaccharide solution, 100 μL PMP (0.5 M) and 50 μL sodium hydroxide (0.3 M) were mixed and incubated at 70 ℃ for 90 min. After adjusting pH to 7.0 with 0.3 M hydrogen chloride (HCl), 1 mL chloroform was added to the mixture. The bottom chloroform layer was removed using a pipe and the top aqueous layer was harvested for HPLC analysis. PMP-labeled polysaccharide was analyzed using an Agilent technologies 1260 series (Agilent Co. USA) which was equipped with diode array detector (DAD) detectors and Agilent ZORBAX SB-C18 column (250 mm × 4.6 mm). The mobile phase was NaH_2_PO_4_/Na_2_HPO_4_ buffer (pH 6.8) and acetonitrile (v/v, 82:18). The flow rate was 1 mL/min. UV absorbance of effluent was monitored at 245 nm.

### Methylation and chromatography-mass spectrometer (GC–MS) analysis

Methylation analysis of polysaccharide was carried out. 5 mg polysaccharide was dissolved in 1.5 mL dimethyl sulfoxide (DMSO) with the addition of 100 mg NaOH. The polysaccharide was methylated by adding 1.5 mL methyl iodide (CH_3_I) followed by a stir for 4 h under a nitrogen protection. The redundant methyl iodide was decomposed by adding deionized water. The methylated polysaccharide was extracted with trichloromethane and evaporated to dryness. Dry methylated polysaccharide was hydrolyzed with 4 M TFA at 100 ℃ for 4 h, reduced with sodium borodeuteride (NaBD_4_) overnight and acetylated with pyridine and acetic anhydride at 100 ℃ for 1 h. Deionized water and dichloromethane were added into the acetylated derivatives. The organic phase was dried under a nitrogen protection. The dry product was dissolved in dichloromethane and analyzed by GC–MS. Trimethylsilylated derivatives were analyzed by HP 7890/5975C GC–MS system (Agilent Technologies Inc. USA) which was equipped with an iron trap MS detector and HP-5MS quartz capillary column (30 mm × 0.25 mm). The temperature program was set as follows: the column at 150 ℃ initial temperature was heated to 200 ℃ at 2 ℃/min, increased to 240 ℃ at 5 ℃/min and held for 5 min. The injection temperature was 230 ℃. The ion source of mass spectrometer was set at 240 ℃.

### Ultraviolet (UV), Infrared Spectroscopy (IR) and NMR spectra analysis

Ultraviolet spectrum of polysaccharide (2 mg/mL) was analyzed by Shimadzu UV-2700 UV–vis spectrophotometer (Shimadzu, Japan) in a wavelength range of 190–600 nm. FT-IR spectrum was determined using fourier transform infrared spectrophotometer (FT-IR) (Nicolet iS10, Thermo Fisher Scientific Inc. America). Background was collected before every sample and the spectra were recorded from 32 scans at 4.00 cm^−1^ resolution with wave numbers ranging of 4000–400 cm^−1^. ^1^H and ^13^C NMR spectra were detected by Bruker Avance spectrometer of 600 or 800 MHz (Germany). The sample was pre-dissolved in deuterium (D_2_O, 99.9%) and lyophilized three times to replace exchangeable protons with D_2_O. All spectra were recorded with HOD suppression by presaturation. The interpretations of ^1^H/^1^H correlated spectroscopy (COSY), total correlation spectroscopy (TOCSY), ^1^H/^13^C heteronuclear single-quantum coherence (HSQC) and heteronuclear multiple bond coherence (HMBC) spectra were analyzed using a state-time proportion phase incrementation for quadrature detection in indirect dimension.

### Isothermal titration calorimetry (ITC), transmission electron microscopy (TEM) and dynamic light scattering (DLS) assay

ITC experiments were performed using a MicroCal PEAQ-ITC. To determine the interactions between polysaccharide and SARS-CoV-2 RBD protein/HIV Env expression plasmid, the entropy and enthalpy were analyzed by ITC [14]. By titrating 0.108 μM (4.9 mg/mL, 60 μL) RBD protein/3.03 μM (3.75 mg/mL, 60 μL) DNA into 0.029 μM (1.25 mg/ml, 200 μL)/7.06 μM (5 mg/mL, 200 μL) PRBS solution at 25 ℃ in water, we obtained the critical thermodynamic parameters including binding affinity (K), enthalpy changes (ΔH) and binding stoichiometry (N). For TEM and DLS assay, the nanoscale morphology and structure of polysaccharide-based nanovaccines were performed by TEM (Tecnai G2 F20 U-TWIN TEM system, American FEI). 700 ml nanovaccine aqueous solution (pH 7.4) was added into a DTS1070 disposable capillary cell to measure the zeta potential and hydrate size of nanovaccine using Zetasizer Nano ZS (Malvern, UK) at 25 °C. The data were analyzed using the MicroCal PEAQ-ITC Analysis Software provided by the manufacturer and fitted with a single-site binding model. Using DLS detection the stability of the assembly between PRBS and RBD/HIV plasmid at 1/4-day, 1/ -day, 1 day, 2 days and 3 days at 56 °C [15, 16].

### Molecular docking study

The crystal structure of SARS-CoV-2 RBD protein was from PDB database (http://www.rcsb.org/). The PDB ID of SARS-CoV-2 RBD protein is 7JMO (2.359 Å). The docking simulation was carried out using auto dock 4.2 program. The geometry of RBD and polysaccharide was optimized using Avogadro software [17]. The docking was performed using the Lamarkian genetic algorithm (LGA). The number of GA runs was set to 100 and the highest populated cluster with the lowest energy conformation based on the scoring function was selected as the binding mode. Among all possible spatial conformations and interaction patterns, the conformation with the lowest energy was selected for visual analysis using PyMolv1.6. Auto dock 4.2 program was used for docking calculations between HIV DNA and polysaccharide. The structure of polysaccharide was obtained from Chem3D and the crustal structure of DNA (PDB ID 1BNA) was obtained from Protein Data Bank (http://www.rcsb.org/). The geometry of DNA and polysaccharide was optimized using Avogadro software. The docking was performed using the Lamarkian genetic algorithm (LGA). The number of GA runs was set to 100 and the highest populated cluster with lowest energy conformation based on the scoring function was selected as the binding mode. The conformation with the lowest energy was selected for visual analysis using PyMolv1.6.x.

### Cell transfection and western blot analysis

293 T cells were maintained in RPMI-1640 medium (1640, GIBCO) with 10% fetal bovine serum (FBS), 100 U/ml penicillin and 100 μg/ml streptomycin. Transfection of 293 T cells with HIV Env DNA plasmid was performed with lipofectamine 3000 transfection reagent (Invitrogen) according to the manufacturers’ protocols. Following transfection (48 h), cells were harvested by centrifugation at 1100×*g* for 5 min at 4 °C and washed once with 1X PBS. For western blot, cells were lysed, subjected to electrophoresis, and stained with specific monoclonal or polyclonal antibodies as described elsewhere.

### Mouse vaccination

All animal studies were approved by the Animal Ethics Committee of Institute of Medical Biology, Chinese Academy of Medical Sciences and Peking Union Medical College, and executed according to guidelines from the Committee of Welfare and Ethics of Laboratory Animals in Yunnan Province. There are six mouse groups in this study, which are (1) blank group consisting of 100 μL saline per each injection, (2) RBD group consisting of 10 μg RBD protein per each injection, (3) PRBS-RBD nanovaccine consisting of 10 μg RBD protein and 50 μg PRBS per each injection, (4) 100 μL blank plasmid group, (5) HIV DNA vaccine group consisting of 10 μg HIV Env expression plasmid and (6) PRBS-HIV DNA nanovaccine group consisting of 10 μg HIV Env expression plasmid and 50 μg PRBS for each injection. Bal B/c mice with 6–8 weeks were intramuscularly injected three vaccines at an interval of 2 weeks.

### Neutralization assay

50 μL SARS-CoV-2 pseudovirus (Sino Biological) co-incubated with diluted mouse serum samples (1:20,1:100,1:250,1:500) at 37 °C for 1 h, and was added into the culture well of ACE2-expressing 293 T cells (5 × 10^4^ cells) for 24 h. Then lysing the cells using a commercial cell lysis buffer (Promega), 10 μL luciferase substrate (Promega) was added and the relative luciferase activity was determined by the luminometer (Bio-Tech). The HOS-CD4-CCR5/CXCR4 cells (8 × 10^4^ cells/well) were inoculated into 6-well plates; 200 μL HIV pseudovirus (SF162.LS) was added to each well, and incubated at 37 °C for 1 h; DEAE was added to each well at a final concentration of 17 μg/ml, and after culturing for 5 h, then added non-resistant complete DMEM medium to each well to a final volume of 1 ml, and incubated for 72 h. The cells were lysed and the luciferase content was detected. The titer of anti-SARS-CoV-2 and anti-HIV neutralizing antibody was calculated by the 50% inhibitory concentration (IC_50_).

### Enzyme-Linked Immunosorbent Assay (ELISA)

The 96-well plates (Costar) were coated with purified antigen proteins (RBD and HIV Env protein, 0.01 μg/mL) or with PRBS (0.01 μg/mL) in phosphate buffer saline (PBS) buffer overnight at 4 °C. ELISA plates were blocked with 5% bovine serum albumin (BSA) in PBS with 0.05% Tween at 37 °C for 2 h. The serum samples were added into each well and incubated for 1 h at 37 °C. The 96-well plates were added into HRP-labeled antibodies with a 1:5000 dilution against mouse IgG, IgG1, IgG2a, IgG2b and IgG3 (Santa Cruz Biotechnology) for an incubation of 1 h at 37 °C. TMB as a chromogenic substrate (Sigma-Aldrich) was added with 100 μL per each well, and incubated for no more than 5 min, following a stop by adding 25 μL 2 M H_2_SO_4_. The optical density (OD) was quantified at 450 and 630 nm by an ELISA plate reader (Thermo Life Sciences).

### IFN-*γ* enzyme-linked immunespot assay (ELISPOT)

The mouse IFN-γ/ELISPOT assay was carried out using the commercial kit (R&D). The mixture of fresh mouse splenocytes (5 × 10^5^ cells) and RBD/HIV Env epitope peptides (5 μg/mL) were incubated in the 96-well plates pre-coated by IFN-γ capture antibody overnight at 37 °C with 5% CO_2_. The plates were washed three times with PBS consisting of 0.05% Tween 20 buffer and incubated for 2 h with the biotinylated goat-anti-mouse IFN-γ monoclonal antibody at 2 μg/mL. After 1 h incubation with an avidin horseradish peroxidase complex in PBS/0.05% Tween 20 buffer, the plates were washed three times with PBS and incubated with peroxidase substrate AEC for 30 min. The spots in each well were measured with the ELISPOT Reader System (Bio-Rad). An absolute value (SFU) > 20 in one million cells is considered positive.

### Flow cytometry analysis

Spleens are isolated from mice at 1 week after the last injections. Fresh splenocytes are divided into two parts. One part stimulated with RBD/HIV Env epitope pool (5 μg/mL) for 4 − 6 h at 37 °C and 5% CO_2_. The viability of splenocytes was assessed using Zombie NIRIM Fixable Viability Kit, washed by cell staining buffer and blocked with anti-FcR antibodies, and stained with surface antibodies as following: CD3-Billiant Violet 510, CD4-FITC, CD8a-Alexa Fluor 700 (Biolegend, the United States). After 30 min incubation at 4 ℃, splenocytes were washed twice by cell staining buffer, fixed and permeabilized using BD Cytofix/Cytoperm Fixation/Permeabilization Kit. Splenocytes were stained 30 min at 4 ℃ with intracellular antibodies of IFN-γ-APC. The viability of the other splenocytes was assessed using Zombie NIRIM Fixable Viability Kit, washed by PBS and blocked with anti-FcR antibodies, and stained with surface antibodies as following: CD45R-B220, H-2kd-FITC, CD11c-BV605, CD80-PE and CD86-PE/cy7 (Biolegend, the United States). After completing the above staining steps, cells were washed twice by PBS with centrifugation. Cells were resuspended in 200 μL cell staining buffer. All samples were acquired on a BD FACS Fortessa and results were analyzed with the FlowJo software v10.7.1.4.

### Cell culture and viability assay

Macrophage RAW264.7 cell line was from American type culture collection (ATCC), The cells were cultured in 1640 RPMI medium supplemented with 10% fetal bovine serum and 100 U/mL of penicillin and 100 μg/mL of streptomycin at 37 ºC and 5% CO_2_. Polysaccharide with a series of final concentrations (0, 50, 100 and 200 μg/mL) were supplemented into the culture medium of cells and incubated for 24 h. After removing cell-culture medium, the cell viability was detected using the CCK-8 kit (Dojindo Molecular Technologies, Inc.).

### Nitric oxide (NO) assay

NO was measured by determining the content of nitrite in cell culture supernatant by nitrate/nitrite assay. Marcophages (2 × 10^5^ cells) was co-incubated with polysaccharide with different concentration (50, 100 and 200 μg/mL) or LPS (1 μg/mL) in 24-well plate for 24 h. Nitrite in the supernatant, as an indicator of NO production, was quantified by Griess reaction.

### Phagocytosis activity assay

The phagocytosis of macrophages was measured by either neutral red or green fluorescent microspheres assay (Sigma). Macrophages (2 × 10^4^ cells/well) were co-incubated with polysaccharide with different concentration (50, 100 and 200 μg/mL) or LPS (1 μg/mL) in a 96-well plate at 37 ℃ for 24 h. Either 0.075% neutral red (150 μL/well) or 2 μm green fluorescent microspheres were added and incubated for 1 h at 37 ℃. For neutral red assay, 150 μL cell lying solution (ethanol: glacial acetic acid = 1:1) was added into each well and incubated at room temperature for 1 h. The absorbance value was detected in a microplate reader at 540 nm. The phagocyte phagocytosis rate was calculated according to the equation: phagocytosis rate (%) = (AS/A0) × 100%. A0 is the absorbance value in the control group. AS is the absorbance value in the treatment group. The phagocytosis of DC2.4 cells line was measured by green fluorescent microspheres assay. DC2.4 cells (1 × 10^5^ cells/well) were co-cultured with either 50 μg/mL PRBS or 1 μg/mL LPS for 24 h. For green fluorescent microspheres assay, 10 μL of 2 μm green fluorescent microspheres was added into each well and incubated at room temperature for 1 h. The fluorescent intensity was quantified using a BioTek fluorescence analysis system (Agilent).

### TNF-*α*, IL-6 and IL-1*β* production

Macrophages (2 × 10^5^ cells/well) were seeded in 12-well plates and cultured for 24 h with polysaccharide with different concentration (50, 100 and 200 μg/mL) or LPS (1 μg/mL). TNF-α, IL-6 and IL-1β in supernatant samples were quantified by the commercial ELISA kits (R&D).

### Physiological assay

Each mouse was intramuscularly injected PRBS for 30 days (400 μg/kg per each day). Fresh mouse blood samples are harvested. Several physiological indicators in mouse blood, such as hemoglobin, platelet, red blood cells, albumin, urea, uric acid, alanine aminotransferase, aspartate transaminase, creatinine and amylase, are quantified by the blood examination instrument (Coulter-JT) and biochemical detector (Roche cobas 6000).

### Immunohistochemical assay

Each mouse was intramuscularly injected PRBS for 30 days (400 μg/kg per each day). The mouse organs (liver, kidney, spleen, heart and lungs) are harvested and use H&E staining to prepare pathological sections. Pathological sections are diagnosed and imaged using a photo-taking optical microscopy (Leica).

### Statistical analysis

Data were expressed as mean ± SD (standard deviation) or mean ± SEM (standard error of mean) of triplicate determination. Statistical significance was analyzed by one-way analysis of variation (ANOVA) and Student's t test with GraphPad Prism software (GraphPad, San Diego, CA, USA). P < 0.05 were statistically significant.

## Results and discussion

### The preparation and characterization of polysaccharide.

We extracted 250 g crude polysaccharide from the rhizomes of Bletilla striata by a series of operations such as hot water extraction, ethanol precipitation and deproteinization. We further purified the crude product using DEAE sepharose fast flow and sephacryl column chromatography. We quantified the polysaccharide by phenol–sulfuric acid method, and obtained pure polysaccharide without residual proteins and nucleic acids, which was confirmed by Bradford protein analysis (Additional file 1: Table S1) and UV spectrum (Additional file 1: Figure S1).

We identified the homogeneousness and molecular weight of polysaccharide. The single and sharp symmetrical peak in the profile of high-performance gel permeation chromatography (HPGPC) indicated an excellent homogeneousness of polysaccharide (Additional file 1: Figure S2, S3). Its average molecular weight was around 42.4 kDa by calculating the standard dextrans and elution time of polysaccharide. The optical rotation of polysaccharide was − 99.35 by circular dichroism (CD) spectrum assay (Additional file 1: Table S1). We also identified the monosaccharide composition of polysaccharide by quantifying 1-phenyl-3-methyl-5-pyrazolone (PMP) derivatives using HPLC. The pure polysaccharide contained both mannose and glucose with a molar ratio of 3.03:1 (Additional file 1: Figure S4).

We investigated the Fourier transform infrared spectrophotometer (FTIR) spectra of pure polysaccharide (Additional file 1: Figure S5). Its absorption peaks distributed in a range of 4000–400 cm^−1.^ A brand nearby 3362 cm^−1^ indicated the stretching vibration of hydroxyl groups. The weak absorption peak at around 2889 cm^−1^ represented a C–H stretching vibration. The peaks at 1200–1000 cm^−1^ were attributed to stretching vibrations of C–O–C and C–O–H of pyranose ring in the polysaccharide. The peak at 903 cm^−1^ was from the asymmetrical stretching vibration of D-glucose. The bands at 869 and 810 cm^−1^ were the characterization of *β*-mannose in the polysaccharide [18].

We defined the glycosides linkages of polysaccharides by gas chromatography-mass spectrometer (GC–MS) analysis. Two sugar residues of the polysaccharides could be elucidated as → 4)-mannose-(1 → (75.2%) → 4)-glucose-(1 → (24.8%) (Additional file 1: Table S2).

**Fig. 1:**
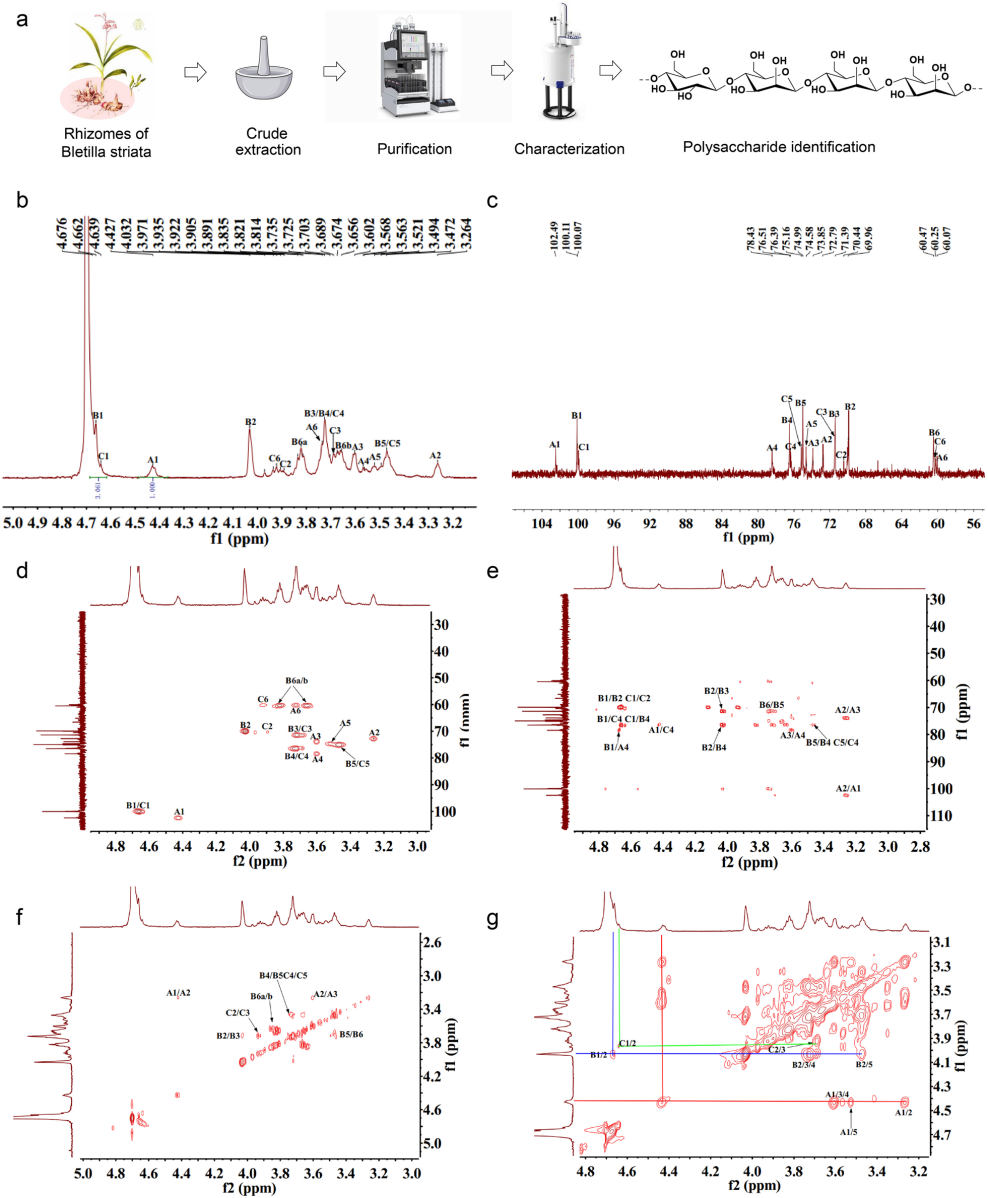
The extraction and identification of polysaccharide. **a** The extraction process and chemical structure of PRBS. **b-g** Expansions of the NMR spectra of PRBS. The ^1^H NMR **b** and ^13^C NMR **c** of 50 mg PRBS dissolved in D_2_O by NMR of 800 MHz. Glucose and mannose were identified in PRBS sample. **d** Heteronuclear single quantum correlation provided the correlations between a carbon and its attached protons. **e** The heteronuclear multiple-bond correlation (HMBC) provided the correlations to carbons, including non-protonated carbons, two or three bonds away from the proton. **f** ^1^H/^1^H chemical-shift correlation spectroscopy (^1^H-^1^H-COSY) provided proton-proton correlations **g** The ^1^H/^1^H-total correlation spectroscopy (^1^H/^1^H-TOCOSY) provided the correlations from a proton to other protons within the same proton spin system

We used nuclear magnetic resonance (NMR) to analyze polysaccharide structure. ^1^H NMR spectrum showed three chemical shifts of anomeric protons at *δ*_H_ 4.42, 4.63 and 4.66 ppm (Fig. 1b). ^13^C NMR spectra showed three anomeric carbon signals at *δ*_C_ 102.48, 100.10 and 99.94 ppm (Fig. 1c). The carbon signals at *δ*_C_ 100.10, 76.51, 74.98, 71.40, 69.89 and 60.47 ppm were higher than others′ apparently. In the heteronuclear single quantum correlation spectrum of BRPS (Fig. 1d), three cross-peaks were found in the anomeric signal area at *δ*4.42/102.48, 4.63/99.94 and 4.66/100.10 ppm. Based on the data from GC–MS and NMR spectra (Additional file 1: Table S2 and Fig. 1b-g), indicated the chemical structure of PRBS may consist of four types of monosaccharide residues, → 4)-*β*-D-glucose-(1 → (A), → 4)-*β*-D-mannose-(1 → (B × 2) and → 4)*-β*-D-mannose-(1 → (C). We identified the sequence of the sugar residues by heteronuclear multiple-bond correlation (HMBC) spectrum (Fig. 1e). Cross-peak signals at *δ* 4.42/76.41 (A H-1/C C-4), 4.63/76.51 (C H-1/B C-4) and 3.72/100.10 (C H-4/B C-1) indicated that *O*-1 of residue A and *O*-1 of residue C were linked to C-4 of residue C and C-4 of residue B. The cross-peaks between H-4 (*δ*_H_ 3.56 ppm) of residue A and C-1 (*δ*_C_ 100.10 ppm) of residue B (A H-4/B C-1) indicated that *O*-1 of residue B was linked to C-4 of residue A. The assignments of proton and carbon resonances for each glycosidic linkage were listed in Additional file 1: Table S3. Taken together, we showed the detailed chemical structure of polysaccharide which possessed an average molecular weight of 42.4 kDa with a linkage of → 4)-*β*-D-glucose-(1 → (A), → 4)-*β*-D-mannose-(1 → (C), → 4)-*β*-D-mannose-(1 → (B), → 4)-*β*-D-mannose-(1 → (B) (Fig. 1a).

### The assembly of polysaccharide-based nanovaccines

**Fig. 2:**
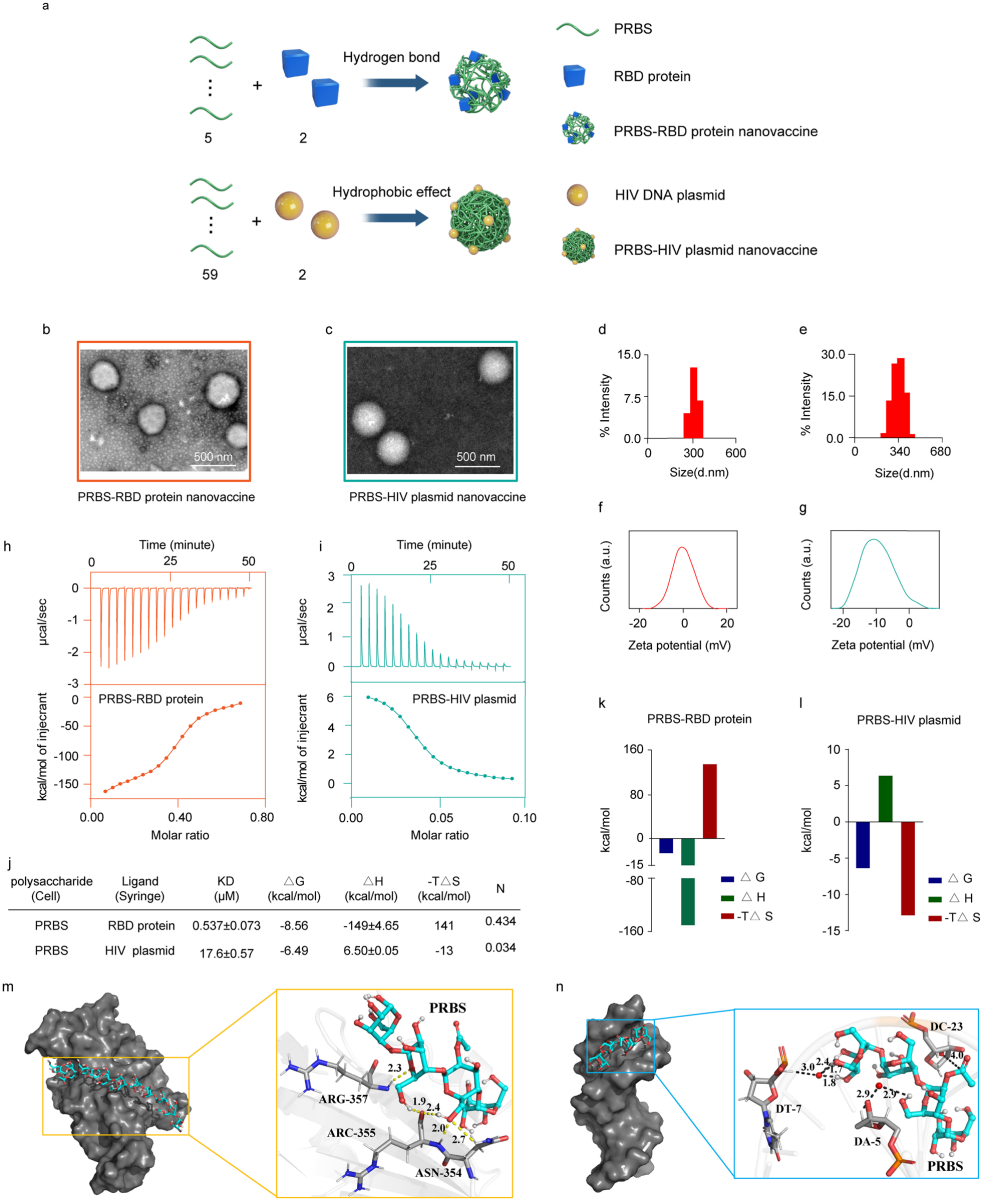
The assembly and characterization of nanovaccines.** a** Schematic diagram for the assembly of nanovaccines. In the assembly of nanovaccine, the molecular ratio between PRBS and RBD protein was 5:2. The molecular ratio between PRBS and HIV plasmid was 59:2. **b** TEM images of PRBS-RBD protein nanovaccine. **c** TEM images of PRBS-HIV plasmid nanovaccine. **d** DLS exhibited that the hydrate size of PRBS-RBD protein nanovaccine was around 300 nm **e** DLS exhibited that the hydrate size of PRBS-HIV plasmid nanovaccine was around 350 nm. **f** The average zeta potential of PRBS-RBD protein nanovaccine was 0 mV. **g** The average zeta potential of PRBS-HIV plasmid nanovaccine was -10 mV. **h** Standard calorimetric titrations of 0.108 μM RBD protein into 0.029 μM PRBS solution at 25 ℃. **i** Standard calorimetric titrations of 3.03 μM HIV DNA plasmid into 7.06 μM PRBS solution at 25 ℃. **j** The thermodynamic parameters of PRBS-RBD protein nanovaccine and PRBS-HIV plasmid nanovaccine such as equilibrium dissociation constant (Kd), Gibbs free energy (ΔG), enthalpy changes (ΔH), entropy changes (ΔS) and binding stoichiometry (N). (k) The fitting data of ITC from PRBS-RBD protein nanovaccine. ΔG, ΔH, − TΔS represented Gibbs free energy of binding (blue), enthalpy changes (green), entropy changes (red). **l** The fitting data of ITC from PRBS-HIV plasmid nanovaccine. ΔG, ΔH, − TΔS represented Gibbs free energy of binding (blue), enthalpy changes (green), entropy changes (red). **m** The structure mode of PRBS-RBD protein nanovaccine. Left part: RBD and PRBS were marked by grey and cyan. Right part: PRBS was depicted by sticks (C, cyan; N, blue; O, red and H, hydrogen). Residues were depicted by sticks (C, royal blue; N, blue; O, red and H, hydrogen). Hydrogen bonds were shown by yellow dotted lines. The length of bond (Å) was shown in the figure. **n** The structure mode of PRBS-HIV plasmid nanovaccine. Left part: HIV plasmid and PRBS were marked by grey and cyan. Right part: PRBS was depicted by sticks (C, cyan; N, blue; O, red and H, hydrogen). The nucleotides of HIV plasmid were depicted by sticks (C, royal blue; N, blue; O, red and H, hydrogen). The location of hydrophobic interaction was shown by a red dot. Water bridges were shown as black dotted lines. The length of bond (Å) was shown in the figure

SARS-CoV-2 RBD protein and HIV Env expression plasmid were selected in this study (Fig. 2a). 15 μg PRBS was gently mixed with either 4 μg RBD protein or 1.5 μg HIV plasmid for 30 min. By transmission electron microscopy (TEM), we observed uniform nanospheres in the mixture of PRBS and RBD protein (around 250 nm diameter; Fig. 2b) and in the mixture of PRBS and HIV plasmid (around 300 nm diameter; Fig. 2c), rather than in the solution of naked PRBS, RBD protein or HIV plasmid (Additional file 1: Figure S6). Using dynamic light scattering (DLS), we characterized the hydrated size and zeta potential of these nanovaccines. The average hydrated size of PRBS-RBD protein and PRBS-HIV plasmid nanovaccine was 300 and 350 nm, separately (Fig. 2d, e). The data from DLS showed that hydrate particle size of the assembly between PRBS and RBD/HIV plasmid have no significant changes at 56 ℃ for 3 days (Additional file 1: Figure S7). The average zeta potential of PRBS-RBD protein and PRBS-HIV plasmid nanovaccine was 0 mV and -10 mV (Fig. 2f, g).

We quantified the intermolecular interactions between PRBS and RBD protein/HIV plasmid by isothermal titration calorimetry (ITC) assay. By titrating 29.0 μM and 7.06 mM PRBS into the solution of 108 μM RBD protein and 3.03 mM HIV plasmid, we detected multiple critical thermodynamic parameters such as equilibrium dissociation constant (Kd), binding stoichiometry (N), Gibbs free energy (ΔG), entropy change (-TΔS) and enthalpy change (ΔH). The Kd values of PRBS-RBD protein and PRBS-HIV plasmid were 0.537 ± 0.073 × 10^−6^ M and 17.6 ± 0.57 × 10^−6^ M (Fig. 2h, i), demonstrating the intermolecular interactions between PRBS and RBD protein/HIV plasmid. Based on the value of binding stoichiometry, we calculated that each RBD protein (N = 0.434) and HIV plasmid (N = 0.034) approximately bind with 2.5 and 29.5 PRBS molecules to form an intermediate (Fig. 2j).

We investigated the dominant intermolecular force to drive the assembly between PRBS and RBD protein/HIV plasmid. In the co-assembly system of PRBS and RBD protein, ΔH value was negative (− 149 kcal/mol) and -TΔS value was positive (141 kcal/mol). This result indicated that the co-assembly between PRBS and RBD protein was enthalpy driven primarily by hydrogen bonds. In the co-assembly system of PRBS and HIV plasmid, ΔH was positive (6.50 kcal/mol) and -TΔS value was negative (− 13 kcal/mol). This result indicated that hydrophobic effects dominantly drove the co-assembly between PRBS and HIV plasmid. Moreover, the negative ΔG value (− 8.56 kcal/mol for PRBS-RBD protein, − 6.49 kcal/mol for PRBS-HIV plasmid) demonstrated that PRBS-based nanovaccines were formulated via a spontaneous assembly manner (Fig. 2k, l).

We further verified the assembly between PRBS and RBD protein/HIV plasmid by stoichiometric calculation. The crystal structure of RBD protein was acquired from PDB database (http://www.rcsb.org/). A detailed analysis of the interaction between PRBS and RBD protein was performed through molecular docking. There were seven hydrogen bonds between the active site of RBD (TYR-473, ARG-457, LEU-455, and LYS-417) and PRBS segments (Fig. 2m and Additional file 1: Table S4). Moreover, the theoretically calculated dock score value of PRBS-RBD protein (− 8.21 kcal/mol) was consistent with that measured by ITC (− 8.56 kcal/mol).

For the co-assembly between PRBS and HIV plasmid (Fig. 2n), we calculated PRBS binds with deoxyadenine-6 (DA-5), deoxyguanine (DG-22), and deoxycytosine-23 (DC-23) nucleotide in the minor groove of HIV plasmid (Additional file 1: Figure S8). Hydrogen bonds were absent of the interactions between PRBS and DA-6/DG-22/DC-23 nucleotide. The co-assembly of PRBS-HIV plasmid was stabilized by two hydrophobic interactions (with DG-22 and DC-23) and two water bridges. The theoretically calculated dock score value of PRBS-HIV plasmid (− 6.20 kcal/mol) was also consistent with that measured by ITC (− 6.49 kcal/mol).

### The immune responses induced by polysaccharide-based nanovaccines

**Fig. 3:**
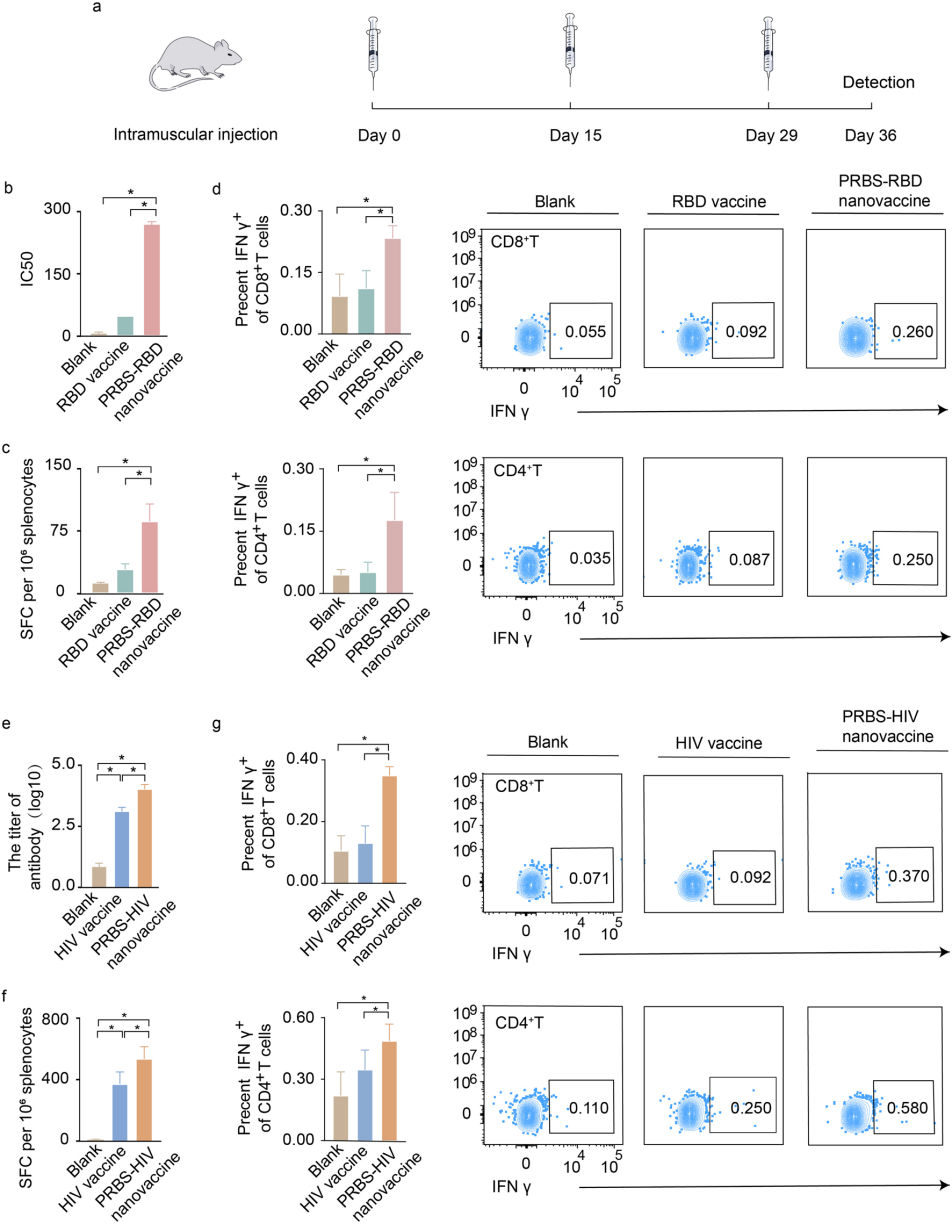
Immune responses induced by PRBS-based nanovaccines. **a** Schematic diagram of immunization procedure. Each mouse received three immunizations with an interval of two weeks. After one week of the final immunization, mice were sacrificed for the immunological assay. **b**The 50% inhibitory concentration (IC_50_) of anti-SARS-CoV-2 neutralizating antibodies induced by either PRBS-RBD protein nanovaccine (10 μg RBD protein and 50 μg PRBS per each injection) or traditional RBD protein vaccine (10 μg RBD protein per each injection). **c** The magnitude of RBD-specific T cell response (IFN-γ ELISPOT) induced by either PRBS-RBD protein nanovaccine (10 μg RBD protein and 50 μg PRBS per each injection) or traditional RBD protein vaccine (10 μg RBD protein per each injection). **d** Using flow cytometry analysis, we assess the secretion of IFN-γ by both CD8^+^T cells and CD4^+^T cells in PRBS-RBD protein nanovaccine group (10 μg RBD protein and 50 μg PRBS per each injection) and traditional RBD protein vaccine group (10 μg RBD protein per each injection). **e** The titer of HIV-specific IgG induced by either PRBS-HIV plasmid nanovaccine (10 μg HIV plasmid and 50 μg PRBS per each injection) or traditional HIV DNA vaccine (10 μg HIV plasmid per each injection). **f** The magnitude of HIV-specific T cell response (IFN-γ ELISPOT) induced by either PRBS-HIV plasmid nanovaccine (10 μg HIV plasmid and 50 μg PRBS per each injection) or traditional HIV DNA vaccine (10 μg HIV plasmid per each injection). **g** CD8^+^ and CD4^+^ T cells IFN-γ response induced by either PRBS-HIV plasmid nanovaccine (10 μg HIV plasmid and 50 μg PRBS per each injection) or traditional HIV plasmid vaccine (10 μg HIV plasmid per each injection). All values are expressed as mean ± SD. *P < 0.05

We evaluated the immune responses induced by PRBS-RBD protein nanovaccine in mice (Fig. 3a). We designed three mouse vaccination groups, which were blank group (100 μL saline per each injection), RBD group (10 μg RBD protein per each injection) and PRBS-RBD protein nanovaccine (10 μg RBD protein and 50 μg PRBS per each injection). There are six mice in each group. All mice received three intramuscular injections with an interval of two weeks. For SARS-CoV-2 vaccines, inducing a potent neutralizing antibody response is critical to block virus infection. Here, we focused on evaluating the capacity of PRBS-RBD protein nanovaccine to produce anti-SARS-CoV-2 neutralizing antibodies. The data from pseudo-virus evaluation system showed that the half-maximal inhibitory concentration (IC_50_) of anti-SARS-CoV-2 neutralizing antibodies induced by PRBS-RBD protein nanovaccine was 5 folds stronger than that induced by traditional RBD protein vaccine (Fig. 3b, Additional file 1: Figure S9). Interestingly, although PRBS-RBD protein nanovaccine induced stronger neutralizing antibodies, the whole magnitude of RBD-specific antibody responses induced by PRBS-RBD protein nanovaccine was similar with that induced by traditional RBD protein vaccine (Additional file 1: Figure S10). This result suggested that PRBS-RBD protein nanovaccine may tend to up-regulate the ratio of neutralizing antibodies among the whole antibodies to obtain an enhanced anti-SARS-CoV-2 neutralizing capacity. To further understand which subtype of IgG majorly neutralizes SARS-CoV-2, we quantified all four subtypes of RBD-specific IgG (IgG1, IgG2a, IgG2b, IgG3) in mice. PRBS-RBD protein nanovaccine induced a significantly enhanced IgG1 response, rather than other three IgG subtype responses, in comparison with traditional RBD protein vaccine (Additional file 1: Figure S11). In line with our results, several previous studies had also reported that IgG1 is the major IgG subtype to neutralize virus infection [19]. Together, this result suggested that PRBS-RBD protein nanovaccine-induced IgG1 may be the critical antibody subtype to neutralize the infection of SARS-CoV-2. We also evaluated the magnitude of PRBS-RBD protein nanovaccine-induced T cell response by IFN-γ ELISPOT assay, which was around 3 folds stronger than that induced by traditional RBD protein vaccine (Fig. 3c, Additional file 1: Figure S12). By flow cytometry, we further verified that PRBS-RBD protein nanovaccine can simultaneously enhance both CD8^+^T and CD4^+^T cell IFN-γ responses than traditional RBD protein vaccine (Fig. 3d, Additional file 1: Figure S13). Taken together, PRBS-RBD protein nanovaccine can significantly improve both neutralizing antibody response and T cell response against SARS-CoV-2 in vivo.

We evaluated immune responses induced by PRBS-HIV plasmid nanovaccine in mice (Fig. 3a). We carried out three intramuscular injections with an interval of two weeks. Mice received 100 μL saline (blank), 10 μg HIV Env expression plasmid (HIV DNA vaccine) and 10 μg HIV Env expression plasmid and 50 μg PRBS (PRBS-HIV plasmid nanovaccine). PRBS-HIV plasmid nanovaccine can express HIV Env antigenic protein (Additional file 1: Figure S14). Previous lessons and experiences showed that a balanced antibody and T cell response was important for improving HIV control in *vivo* [20]. HIV-specific IgG elicited by PRBS-HIV plasmid nanovaccine was significantly stronger than that induced by traditional HIV DNA vaccine in mice (P < 0.05; Fig. 3e). Further, we evaluated the capacity of PRBS-HIV plasmid nanovaccine to produce anti-HIV neutralizing antibodies. The data from pseudo-virus evaluation system presented that the half-maximal inhibitory concentration (IC_50_) of anti-HIV neutralizing antibodies induced by PRBS-HIV plasmid nanovaccine was stronger than that induced by traditional HIV DNA vaccine (Additional file 1: Figure S15). The magnitude of HIV-specific IFN-γ cellular response induced by PRBS-HIV plasmid nanovaccine was also significantly higher than that induced by traditional HIV DNA vaccine (P < 0.05; Fig. 3f, Additional file 1: Figure S16). Further investigation showed that PRBS-HIV plasmid nanovaccine significantly enhanced both CD8^+^T and CD4^+^T cell IFN-γ responses (Fig. 3g, Additional file 1: Figure S13). Taken together, PRBS-HIV plasmid nanovaccine enhanced both antibody and T cell responses in mice, therefore exhibiting a promising potential for improving HIV control in vivo.

### The immunoregulation of PRBS on the behaviors and functions of immune cells

**Fig. 4:**
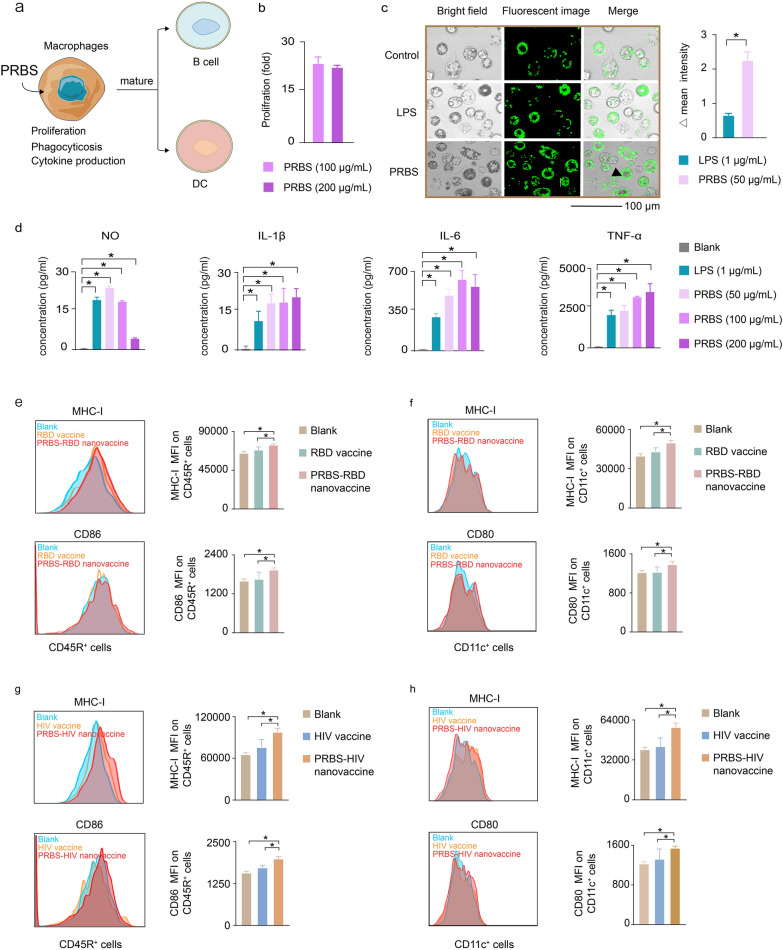
The immunoregulation of PRBS on the behaviors and functions of macrophages. **a** Schematic illustration: the effect of PRBS in regulating the behaviors and functions of macrophages. PRBS can promote the proliferation of macrophages, enhance the phagocytic ability of macrophages and increase the secretion of bioactive molecules from macrophages. **b** PRBS (100 μg/mL and 200 μg/mL) dramatically promoted the proliferation of macrophages. **c** Fluorescence images for the phagocytosis of macrophages. Saline and 1 μg/mL lipopolysaccharide (LPS, a positive stimulator) was used as negative control and positive control. The value of △mean intensity was obtained according to the formula: The value of △mean intensity = (The value of fluorescence intensity from test samples—The value of fluorescence intensity from negative control samples) × 100%. **d** The production of bioactive molecules (NO, IL-1β, IL-6, TNF-α) from macrophages. Saline and 1 μg/mL LPS were used as negative control and positive control. **e** The expression of MHC-I and CD86 on B cells (CD45R^+^) from either PRBS-RBD protein nanovaccine group (10 μg RBD protein and 50 μg PRBS per each injection) or traditional RBD protein vaccine group (10 μg RBD protein per each injection). **f** The expression of MHC-I and CD80 on DC (CD11c^+^) from either PRBS-RBD protein nanovaccine group (10 μg RBD protein and 50 μg PRBS per each injection) or traditional RBD protein vaccine group (10 μg RBD protein per each injection). **g** The expression of MHC-I and CD86 on B cells (CD45R^+^) from either PRBS-HIV plasmid nanovaccine group (10 μg HIV plasmid and 50 μg PRBS per each injection) or traditional HIV plasmid vaccine group (10 μg HIV plasmid per each injection). **h** The expression of MHC-I and CD80 on DC (CD11c^+^) from either PRBS-HIV plasmid nanovaccine group (10 μg HIV plasmid and 50 μg PRBS per each injection) or traditional HIV plasmid vaccine group (10 μg HIV plasmid per each injection).All values were expressed as mean ± SD. *P < 0.05

We investigated the effect of PRBS on regulating the behaviours and functions of macrophages. Macrophage is a critical type of immune cells, which not only can effectively internalize and present antigens, but also regulate the functions of various immunologic effector cells such as B cells and T cells [21, 22] (Fig. 4a). We investigated the effect of PRBS on stimulating the proliferation of macrophages. Macrophages were co-incubated with either 100 or 200 μg/mL PRBS for 72 h. The number of macrophages in PRBS-treated group dramatically increased to around 20 folds (Fig. 4b). This result demonstrated that PRBS can potently promote the proliferation of macrophages. Moreover, the phagocytosis capacity of PRBS-treated macrophages was evaluated as following: macrophages were co-cultured with either 50 μg/mL PRBS or 1 μg/mL lipopolysaccharide (LPS, a positive stimulator). After 72 h incubation, PRBS-treated macrophages exhibited around 3 folds stronger phagocytosis capacity against green fluorescent microspheres (2 μm) than LPS-treated macrophages (Fig. 4c). Significantly enhanced phagocytosis capacity of macrophages can also be realized by PRBS treatment in a broad concentration range from 50 to 200 μg/mL (Additional file 1: Figure S11).

We evaluated the activity of PRBS in stimulating macrophages to produce bioactive molecules such as nitric oxide (NO), tumor necrosis factor α (TNF-α), interleukin 6 (IL-6) and interleukin 1β (IL-1β) [23, 24]. NO has a wide distribution in various tissues and organs and plays an important role in modulating both innate and adaptive immunity [25, 26]. Both 50 and 100 mg/mL PRBS can stimulate macrophages to produce significantly higher concentration NO than untreated macrophages. In contrast, 200 μg/mL PRBS did not promote macrophages to produce stronger NO. These results suggested that PRBS may activate macrophages to produce NO by a concentration-dependent manner. We also evaluated the activity of PRBS in stimulating macrophages to secrete various cytokines. TNF-α, IL-6 and IL-1β are three important cytokines for regulating macrophage-mediated immune responses [27, 28].In comparison with untreated macrophages, in a broad PRBS concentration range from 50 to 200 μg/mL, PRBS-stimulated macrophages secreted significantly stronger TNF-α, IL-6 and IL-1β (Fig. 4d).

Activated macrophages-derived C3d can bind with B cell receptor (BCR) and B cell activation co-receptor (CD21/CD19/CD81/CD225 complex) to promote the maturation B cells [29]. Macrophages-secreted cytokines such as TNF-α, IL-6 and IL-1β can strengthen the expressions of MHC molecules and B7 family molecules (CD80 and CD86) on the surface of dendritic cells (DCs) to facilitate the differentiation from immature DCs to mature DCs [30]. Besides, we co-cultured DCs with either 50 μg/mL PRBS or 1 μg/mL LPS. After 24 h, PRBS-treated DCs displayed the stronger phagocytosis against green fluorescent microspheres (2 μm) than LPS-treated DCs (Additional file 1: Figure S20). Since these important roles of macrophages in stimulating the maturation of B cells and DCs [31], we further investigated whether PRBS-based nanovaccines can activate macrophages to mature B cells and DCs in vivo. For SARS-CoV-2 protein vaccine, the expressions of MCH-I and CD86 on B cells from PRBS-RBD protein nanovaccine-injected mice were significantly higher than those from traditional RBD protein vaccine-injected mice (Fig. 4e, Additional file 1: Figure S17), suggesting that PRBS-RBD protein nanovaccine possibly promoted B cell maturation to boost anti-SARS-CoV-2 neutralizing antibody response. PRBS-RBD protein nanovaccine also significantly increased the expressions of MCH-I and CD80 on DCs, indicating the enhancement of antigen presentation of PRBS-RBD protein nanovaccine (Fig. 4f, Additional file 1: Figure S17). Similarly, for HIV DNA vaccine, the expressions of MCH-I and CD86 on B cells (Fig. 4g, Additional file 1: Figure S18) and the expressions of MCH-I and CD80 on DCs (Fig. 4h, Additional file 1: Figure S18) from PRBS-HIV plasmid nanovaccine-injected mice were also significantly higher than those from traditional HIV plasmid vaccine-injected mice. These results suggested that PRBS-HIV nanoplasmid vaccine can promote B cell maturation and antigen presentation to elicit potent antibody and cellular immune response.

Taken together, we demonstrated the effect of PRBS on activating multiple types of critical immune cells such as macrophages, B cells and DCs. These findings provided a convincing evidence to explain why PRBS-based nanovaccines can elicit potent antigen-specific immune responses.

### The biocompatibility and biosafety of PRBS

**Fig. 5:**
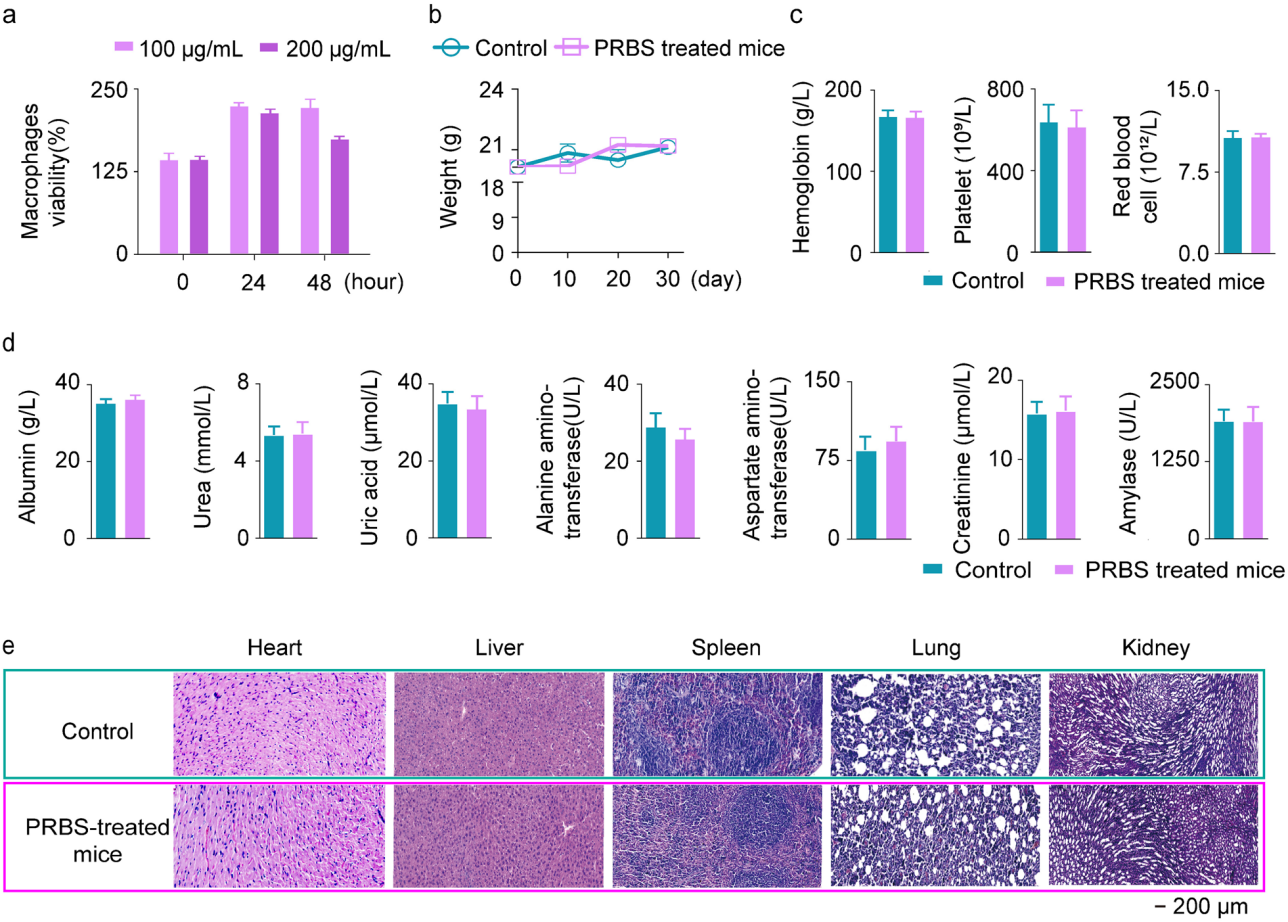
The biocompatibility and biosafety of PRBS. **a** The viability of macrophages treated with 100 μg/mL and 200 μg/mL PRBS at 0, 24, 48 h incubation. **b** The weight of mice in control group (normal mice) and PRBS-treated group (400 μg/kg PRBS). **c** The concentration of hemoglobin, the number of platelet and red blood cells in control group (normal mice) and PRBS-treated group (400 μg/kg PRBS). **d** The concentrations of albumin, urea, uric acid, alanine aminotransferase, aspartate transaminase, creatinine and amylase in mouse serum samples from control group (normal mice) and PRBS-treated group (400 μg/kg PRBS). **e** The immunohistochemical images (H&E staining) of heart, liver, spleen, lung and kidney from control group (normal mice) and PRBS-treated group (400 μg/kg PRBS). Scale bar = 200 μm

We evaluated the biocompatibility and biosafety of PRBS*.* Both medium and high concentration of PRBS (100 and 200 μg/mL) did not cause the cytotoxicity against the viability of macrophages after 0, 24, 48 h of incubation in vitro (Fig. 5a). In comparison with normal mice, 200 μg/mL PRBS did not affect the growth of mouse weight (Fig. 5b). The concentration of hemoglobin, the number of platelet and red blood cells in PRBS-treated mice were also consistent with those in normal mice (Fig. 5c). Multiple important physiological indicators, such as albumin, urea, uric acid, alanine aminotransferase, aspartate transaminase, creatinine and amylase, did not show significant differences between PRBS-treated mice and normal mice (Fig. 5d). Moreover, immunohistochemical analysis from heart, liver, spleen, lung and kidney exhibited neither infiltration of inflammatory cells nor necrosis of organ tissue in PRBS-treated mice (Fig. 5e). All these results indicated that PRBS possessed a satisfactory biocompatibility and biosafety.

## Conclusion

Natural polysaccharides distribute in almost all of organisms from prokaryotes to eukaryotes and act as the critical components to participate in all kinds of life activities [32, 33]. For decades, researchers have devoted to use natural polysaccharides as building blocks to construct diverse functional biomaterials [34–36]. Amounts of active groups (hydroxyl, amidogen and sulfydryl) can endow polysaccharides with convenient modifications on the surface or the interior of bio-scaffolds to formulate various functional derivatives for the applications [37, 38] of soft tissue engineering engineering [39, 40] and delivery carriers [41–43]. Moreover, most of polysaccharides-based constructions or modifications have been performed in mild and nontoxic aqueous environments. All these properties potentially make polysaccharides well suited for formulating various functional biomaterials.

In the current study, we identified a new natural polysaccharide from the rhizomes of Bletilla striata. The results from NMR and FTIR revealed that our natural polysaccharide (PRBS) possessed abundant active groups (-OH). Both RBD protein antigen and HIV DNA antigen can interact with these active groups on PRBS by hydrogen bond and hydrophobic effect to provide assembly driving force. PRBS has not or very poor inherent immunogenicity, because PRBS-specific antibodies are undetectable in the PRBS-injected mice (Additional file 1: Figure S21). Moreover, our data (Additional file 1: Figure S19) showed the potent immunostimulation effect on enhancing the phagocytosis of macrophages, which is critical for promoting antigen presentation and inducing potent responses [31]. Together, these results support our starting hypothesis, in which our natural polysaccharide (PRBS) is a potential platform candidate for constructing self-adjuvant nanovaccines.

Next, we used this polysaccharide as a versatile platform to construct polysaccharide-protein nanovaccine against SARA-CoV-2 and polysaccharide-DNA nanovaccine against HIV. These nanovaccines can significantly enhance both antibody and T cell immune responses without the addition of adjuvants, in comparison to their traditional forms in *vivo*. Actually, we have used Alu and CpG as the control. PRBS showed a similar effect in enhancing humoral and cellular response with Alu/CpG (Additional file 1: Figure S22). In conclusion, our study set an example to construct the powerful self-adjuvant nanovaccines based on the polysaccharide platform.

To understand the assembly mechanism of polysaccharide-based nanovaccines, the roles of intermolecular forces in driving the co-assembly between polysaccharides and other types of molecules have been revealed. To the best of our knowledge, several intermolecular forces, such as hydrophobic effect, hydrogen bonds, electrostatic adsorption, aromatic-aromatic interactions and Vander Waals force, can mediate the co-assembly among homologous or heterogenous molecules. However, the exact role of each intermolecular force in driving the assembly of polysaccharide-based nanovaccines remains poorly understood. Two previous studies shared the importance of electrostatic adsorption in driving the co-assembly between polysaccharides and proteins [44–46]. One convincing explanation was that the quaternary ammonium groups in polysaccharides can provide positive charges to form the assembly with negatively charged proteins. Of note, our study experimentally provided new evidence to demonstrate the importance of hydrogen bonds in driving the co-assembly between polysaccharide and protein. In addition, we experimentally revealed the role of hydrophobic interactions in driving the co-assembly between polysaccharide and DNA. These findings extended our understanding for the assembly between polysaccharides and other types of molecules, therefore benefiting us to rationally design the assembly systems for the construction of polysaccharide-based nanovaccines.

In addition, the polysaccharide (PRBS) in this study can be conveniently prepared with a large quantity. We can formulate > 15 g purified PRBS from around 1 kg rhizomes of Bletilla striata, which exhibited a > 1.5% preparation efficiency. Importantly in comparison with other known assembly strategies such as enzymatic self-assembly and chemical assembly [47–49], PRBS-based self-assembly can avoid the supplement of enzymes or chemical catalysts, therefore simplifying the formulation process of vaccines. The satisfactory biocompatibility and biosafety of PRBS further facilitates its clinical applications. Taken together, all of above advantages highlight the promising potential of PRBS for a final transformation and application in the field of vaccine.

## Supplementary Information

**Additional file 1**: ** Table S1.** Major chemical content of the polysaccharide PRBS. **Table S2.** The results of chemical analyses of PRBS fractions. **Figure S1.** Ultraviolet spectrum of PRBS (2 mg/mL) was analyzed by Shimadzu UV-2700 UV-vis spectrophotometer in the wavelength range of 190-600 nm. **Figure S2.** Polysaccharide purity by high-performance gel permeation chromatography (HPGPC) profiles. **Figure S3.** Standard curve of average molecular weight determination by high-performance gel permeation chromatography (HPGPC) profiles. **Figure S4.** HPLC analysis of monosaccharide standards and PRBS after PMP derivation. **Figure S5.** FT-IR spectra showing the characteristic absorption peaks of PRBS such as hydroxyl and carbony l.20 μL PRBS (2 mg/mL) aqueous solution mixed with KBr powder, dried and pressed, then detected by FT-IR. **Table S3.** Chemical shifts for the resonances of glycosyl residues of PRBS 1H /13C NMR spectra. **Figure S6.** TEM images of naked PRBS, RBD protein and HIV plasmid. **Figure S7.** (a) DLS exhibited that the hydrate size of PRBS-RBD protein nanovaccine on 1/4,1/2,1,2,3 days. (b) DLS exhibited that the hydrate size of PRBS-HIV plasmid nanovaccine on 1/4,1/2,1,2,3 days. **Table S4.** Hydrogen-bonds between PRBS and RBD. **Figure S8.** Intermolecular interactions of the PRBS docked to HIV plasmid. **Figure S9.** The inhibitory concentration curves of anti-SARS-CoV-2 neutralizating antibodies induced by either PRBS-RBD protein nanovaccine (10 μg RBD protein and 50 μg PRBS per each injection) or traditional RBD protein vaccine (10 μg RBD protein per each injection). **Figure S10.** IgG responses in three mouse vaccination groups: Blank group (100 μL saline per each injection), RBD vaccine (10 μg RBD protein per each injection), and 100 μL PRBS-RBD nanovaccine (10 μg RBD protein and 50 μg PRBS). All groups’ data from 1:10000 dilution. **Figure S11.** IgG subtype responses (IgG1, IgG2a, IgG2b, IgG3) in three mouse vaccination groups: Blank group (100 μL saline per each injection), RBD vaccine (10 μg RBD protein per each injection) and 100 μL PRBS-RBD nanovaccine (10 μg RBD protein and 50 μg PRBS). All groups’ data from 1:1000 dilution. All values are expressed as mean ± SEM for three duplicates. *P < 0.05 versus the control group. **Figure S12.** The specific IFN-γ ELISPOT photography results of RBD-specific T cell response induced by either PRBS-RBD protein nanovaccine (10 μg RBD protein and 50 μg PRBS per each injection) or traditional RBD protein vaccine (10 μg RBD protein per each injection). **Figure S13.** The representative gating strategy between parallel groups. **Figure S14.** Western blotting results of HIV env plasmid expressing gp140 protein. **Figure S15.** The IC50 of anti-HIV neutralizing antibodies induced by either PRBS-HIV plasmid nanovaccine (10 μg HIV plasmid and 50 μg PRBS per each injection) or traditional HIV DNA vaccine (10 μg HIV plasmid per each injection). **Figure S16.** The specific IFN-γ ELISPOT photography results of HIV-specific T cell response induced by either PRBS-HIV plasmid nanovaccine (10 μg HIV plasmid and 50 μg PRBS per each injection) or traditional HIV DNA vaccine (10 μg HIV plasmid per each injection). **Figure S17.** The representative detailed gating strategy and parallel groups results from either PRBS-RBD protein nanovaccine group (10 μg RBD protein and 50 μg PRBS per each injection) or traditional RBD protein vaccine group (10 μg RBD protein per each injection). **Figure S18.** The representative detailed gating strategy and parallel groups results from either PRBS-HIV plasmid nanovaccine group (10 μg HIV plasmid and 50 μg PRBS per each injection) or traditional HIV plasmid vaccine group (10 μg HIV plasmid per each injection). **Figure S19.** Different concentrations of PRBS (100 μg/mL or 200 μg/mL) promote the phagocytosis ability of macrophages. **Figure S20.** Fluorescence images for the phagocytosis of dendritic cells (DC2.4). **Figure S21.** PRBS-specific IgG responses in two mouse vaccination groups: PRBS (50 μg PRBS per each injection), and negative control (100 μL salin per each injection). **Figure S22.** (a) IgG responses in two mouse vaccination groups: Alu-RBD vaccine (10 μg RBD protein and 35 μg Alu per each injection), and 100 μL PRBS-RBD nanovaccine (10 μg RBD protein and 50 μg PRBS per each injection). All groups’ data from 1:10000 dilution. (b) The titer of HIV-specific IgG induced by either CpG-HIV vaccine (15 μg CpG and 10 μg HIV plasmid per each injection) or 100 μL PRBS-HIV nanovaccine (10 μg HIV plasmid and 50 μg PRBS per each injection) (c) The magnitude of RBD-specific T cell response (IFN-γ ELISPOT) induced by either Alu-RBD vaccine (10 μg RBD protein and 35 μg Alu per each injection) or 100 μL PRBS-RBD nanovaccine (10 μg RBD protein and 50 μg PRBS per each injection). (d) The magnitude of HIV-specific T cell response (IFN-γ ELISPOT) induced by either CpG-HIV vaccine (15 μg CpG and 10 μg HIV plasmid per each injection) or 100 μL PRBS-HIV nanovaccine (10 μg HIV plasmid and 50 μg PRBS per each injection).

## Data Availability

The data that support the findings of this study are available from the corresponding author upon reasonable author upon reasonable request.

## Abbreviations

PRBS: Polysaccharide from the Rhizomes of Bletilla striata
SARS-CoV-2: Severe Acute Respiratory Syndrome Coronavirus 2
RBD: Receptor Binding Domain
HIV: Human Immunodeficiency Virus
DNA: Deoxyribonucleic Acid
APC: Antigen-Presenting Cell
ITC: Isothermal Titration Calorimetry
DEAE: Dicthylaminoethyl
HPGPC: High Performance Gel Permeation Chromatography
CD: Circular Dichroism
FTIR: Fourier Transform Infrared Spectroscopy
GC–MS: Gas Chromatography-Mass Spectrometer
NMR: Nuclear Magnetic Resonance
TEM: Transmission Electron Microscopy
Ig: Immunoglobulin
ELISA: Enzyme Linked Immunosorbent Assay
IFN-γ: Interferon γ
ELISPOT: Enzyme-Linked Immunosorbent Spot
C3d: Complement 3d
BCR: B cell Receptor
NO: Nitric oxide
TNF-α: Tumor necrosis factor α
IL-6: Interleukin 6
IL-1β: Interleukin 1β
DCs: Dendritic Cells
MCH-I: Major Histocompatibility Complex- I
