# Supplementary material for: A new polysaccharide platform constructs self-adjuvant nanovaccines to enhance immune responses
Source: J Nanobiotechnology. 2022 Jul 14;20:320. doi: 10.1186/s12951-022-01533-3 (PMC9281129; doi:10.1186/s12951-022-01533-3)
Supplement: Supplementary file 1 — Additional file 1: Table S1. Major chemical content of the polysaccharide PRBS. Table S2. The results of chemical analyses of PRBS fractions. Figure S1. Ultraviolet spectrum of PRBS (2 mg/mL) was analyzed by Shimadzu UV-2700 UV-vis spectrophotometer in the wavelength range of 190-600 nm. Figure S2. Polysaccharide purity by high-performance gel permeation chromatography (HPGPC) profiles. Figure S3. Standard curve of average molecular weight determination by high-performance gel permeation chromatography (HPGPC) profiles. Figure S4. HPLC analysis of monosaccharide standards and PRBS after PMP derivation. Figure S5. FT-IR spectra showing the characteristic absorption peaks of PRBS such as hydroxyl and carbony l.20 μL PRBS (2 mg/mL) aqueous solution mixed with KBr powder, dried and pressed, then detected by FT-IR. Table S3. Chemical shifts for the resonances of glycosyl residues of PRBS 1H /13C NMR spectra. Figure S6. TEM images of naked PRBS, RBD protein and HIV plasmid. Figure S7. (a) DLS exhibited that the hydrate size of PRBS-RBD protein nanovaccine on 1/4,1/2,1,2,3 days. (b) DLS exhibited that the hydrate size of PRBS-HIV plasmid nanovaccine on 1/4,1/2,1,2,3 days. Table S4. Hydrogen-bonds between PRBS and RBD. Figure S8. Intermolecular interactions of the PRBS docked to HIV plasmid. Figure S9. The inhibitory concentration curves of anti-SARS-CoV-2 neutralizating antibodies induced by either PRBS-RBD protein nanovaccine (10 μg RBD protein and 50 μg PRBS per each injection) or traditional RBD protein vaccine (10 μg RBD protein per each injection). Figure S10. IgG responses in three mouse vaccination groups: Blank group (100 μL saline per each injection), RBD vaccine (10 μg RBD protein per each injection), and 100 μL PRBS-RBD nanovaccine (10 μg RBD protein and 50 μg PRBS). All groups’ data from 1:10000 dilution. Figure S11. IgG subtype responses (IgG1, IgG2a, IgG2b, IgG3) in three mouse vaccination groups: Blank group (100 μL saline per each injection), RBD vaccine (1 [file 12951_2022_1533_MOESM1_ESM.doc]

Supporting Information

A new polysaccharide platform constructs self-adjuvant nanovaccines to enhance immune responses

**Sisi Chen^1‡^, Liu Yang^2 ‡^, Xia Ou^3 ‡^, Jin-Yu Li^2 ‡^, Cheng-Ting Zi^4^, Hao Wang^5^ *, Jiang-Miao Hu^2^ *, Ye Liu^1^ ***

1 Institute of Medical Biology, Chinese Academy of Medical Sciences and Peking Union Medical College, Kunming, Yunnan, 650000, China.

2 State Key Laboratory of Phytochemistry and Plant Resources in West China, and Yunnan Key Laboratory of Natural Medicinal Chemistry, Kunming Institute of Botany, Chinese Academy of Sciences, Kunming, Yunnan, 650201, China.

3 School of Medicine, Kunming university of Science and Technology, Kunming, Yunnan, 650201, China.

4 College of Science, Yunnan Agricultural University, Kunming, Yunnan, 650201, China.

5 CAS Center for Excellence in Nanoscience, CAS Key Laboratory for Biomedical Effects of Nanomaterials and Nanosafety, National Center for Nanoscience and Technology, Beijing100190 China.

^‡^ Authors equally contribute this work.

* Corresponding authors: [liuye@imbcams.com.cn](mailto:liuye@imbcams.com.cn); [hujiangmiao@mail.kib.ac.cn](mailto:hujiangmiao@mail.kib.ac.cn);

[wanghao@nanoctr.cn](mailto:wanghao@nanoctr.cn).

**Supplementary Tables and Figures**

Table S1. Major chemical content of the polysaccharide PRBS

|  | Carbohydrate content (%) | | Protein content (%) | Molecular weight (kDa) | | Optical rotation [α]21 D | |
| --- | --- | --- | --- | --- | --- | --- | --- |
| PRBS | | 94.2 | － | | 42.4 | | -99.35 |

“－” undetectable

Table S2. The results of chemical analyses of PRBS fractions.

| Methylated sugar | Type of linkage | Molar Ratio % | | Mass Fragment (*m/z*) | |
| --- | --- | --- | --- | --- | --- |
| 2,3,6-Me_3_-mannose | →4)-mannose-(1→ | | 75.2 | | 43, 87, 99, 117, 129, 143, 161, 183, 203, 233 |
| 2,3,6-Me_3_-glucose | →4)-glucose-(1→ | | 24.8 | | 43, 71, 87, 101, 117, 129, 142, 161, 203, 233 |
|  |  | |  | |  |


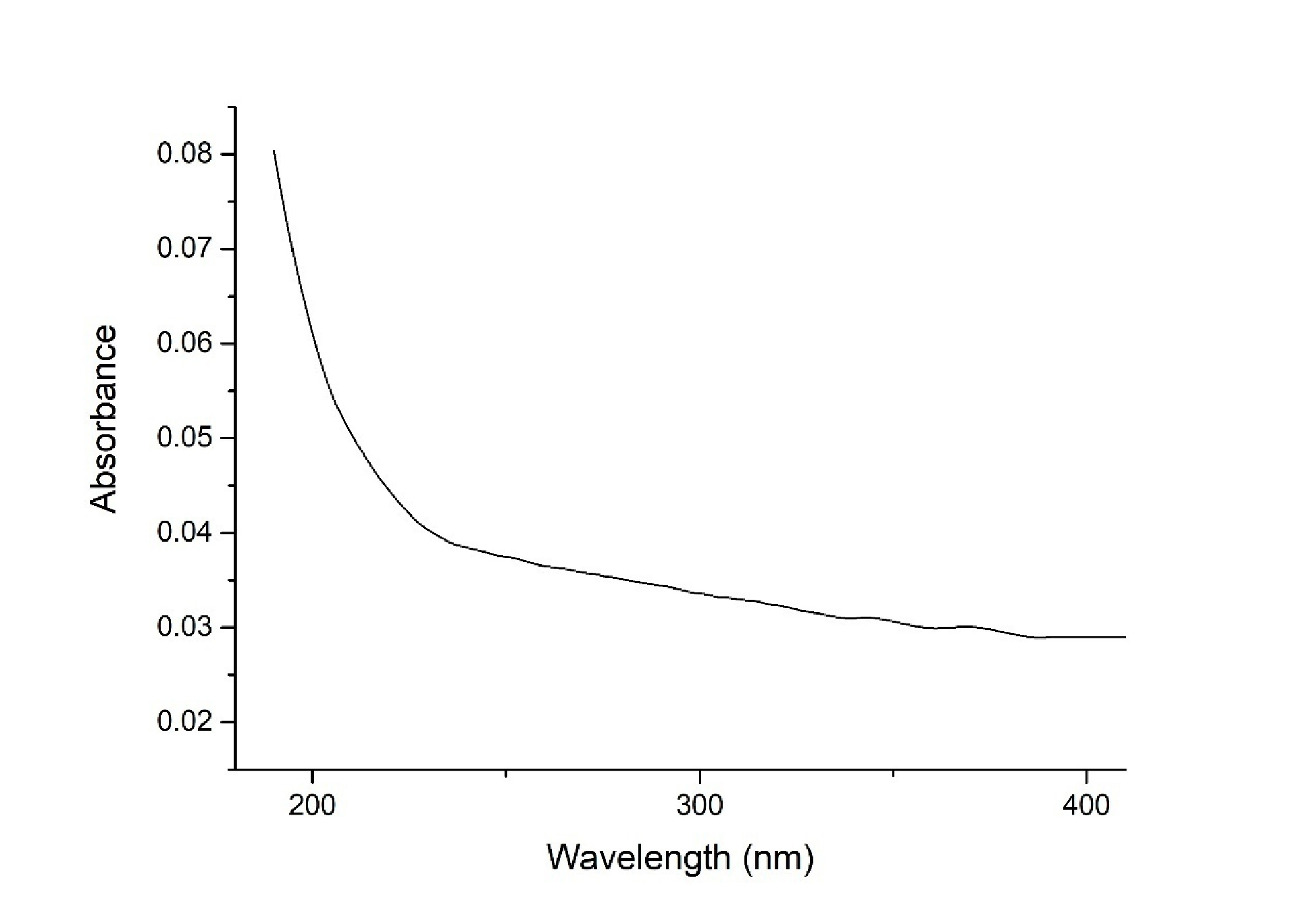


**Figure S1.** Ultraviolet spectrum of PRBS (2 mg/mL) was analyzed by Shimadzu UV-2700 UV-vis spectrophotometer in the wavelength range of 190-600 nm.


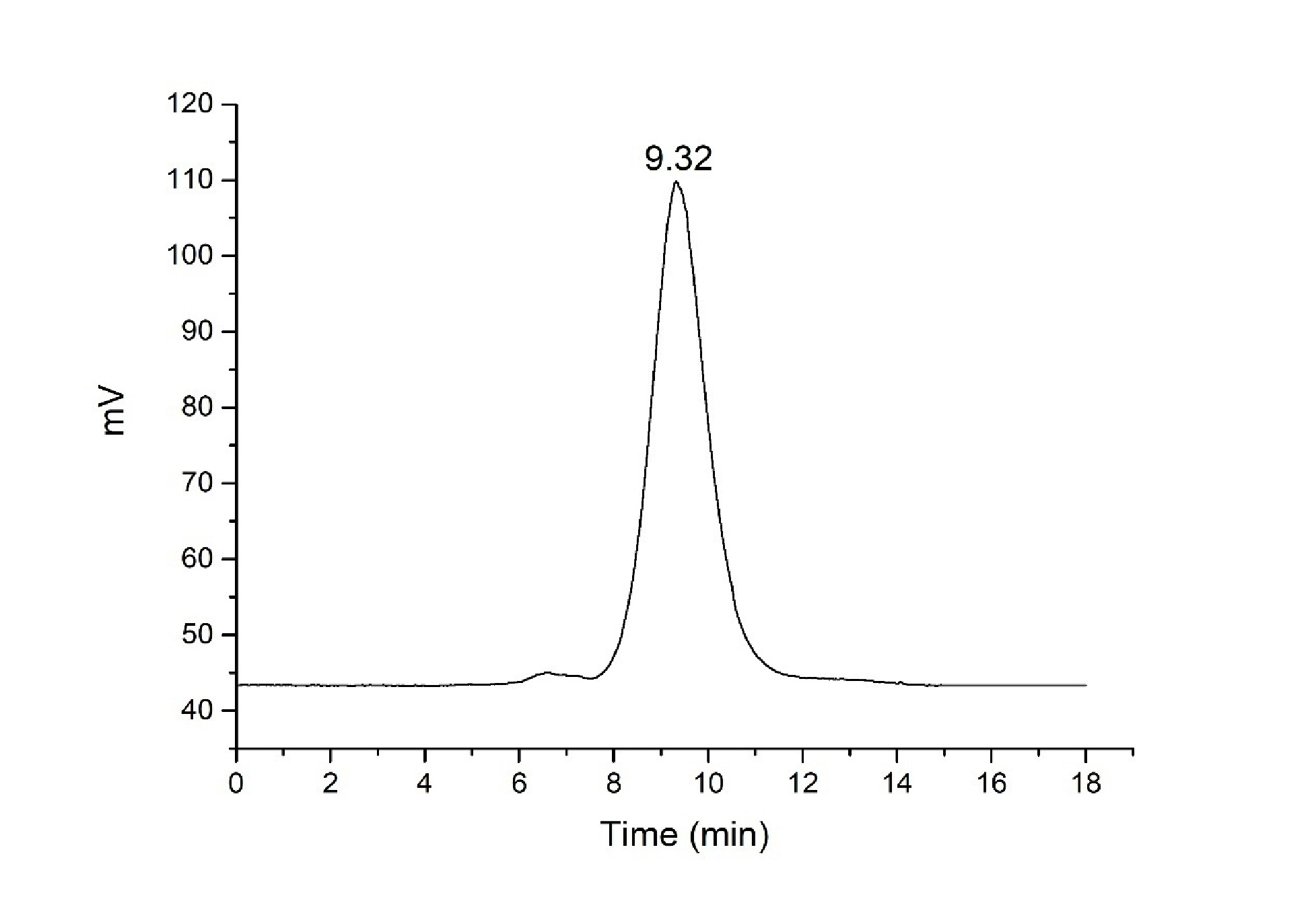


**Figure S2.** Polysaccharide purity by high-performance gel permeation chromatography (HPGPC) profiles. The presence of 1 mg/mL PRBS monitored by HPLC at ELSD. The peaks eluted at 9.32 min represent PRBS. Chromatographic conditions: sample: PRBS 1 mg/mL; chromatographic column: TSKgel-G4000Wxl 7.8 mm*30 cm; mobile phase: 100% water isocratic elution; Elution time: 18 min; Injection volume:10 μL.


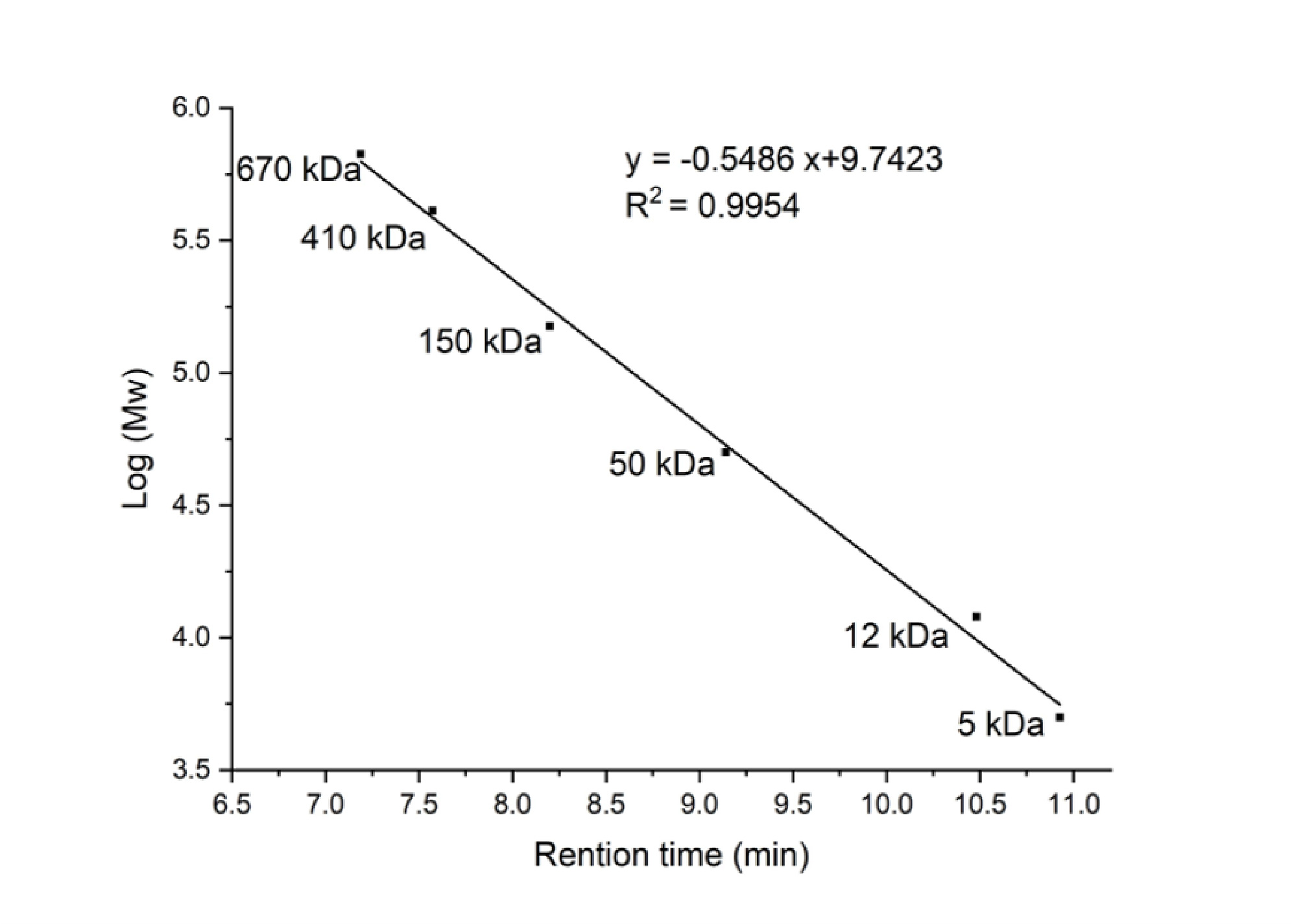


**Figure S3.** Standard curve of average molecular weight determination by high-performance gel permeation chromatography (HPGPC) profiles. Chromatographic conditions: sample: Standard sugars (5, 12, 50, 150, 410, 670 kDa) 1 mg/mL; chromatographic column: TSKgel-G6000Wxl 7.8 mm*30 cm and TSKgel-G4000Wxl 7.8 mm*30 cm; mobile phase: wate 100% isocratic elution; Elution time: 30 min; Injection volume: 10 μL.

**
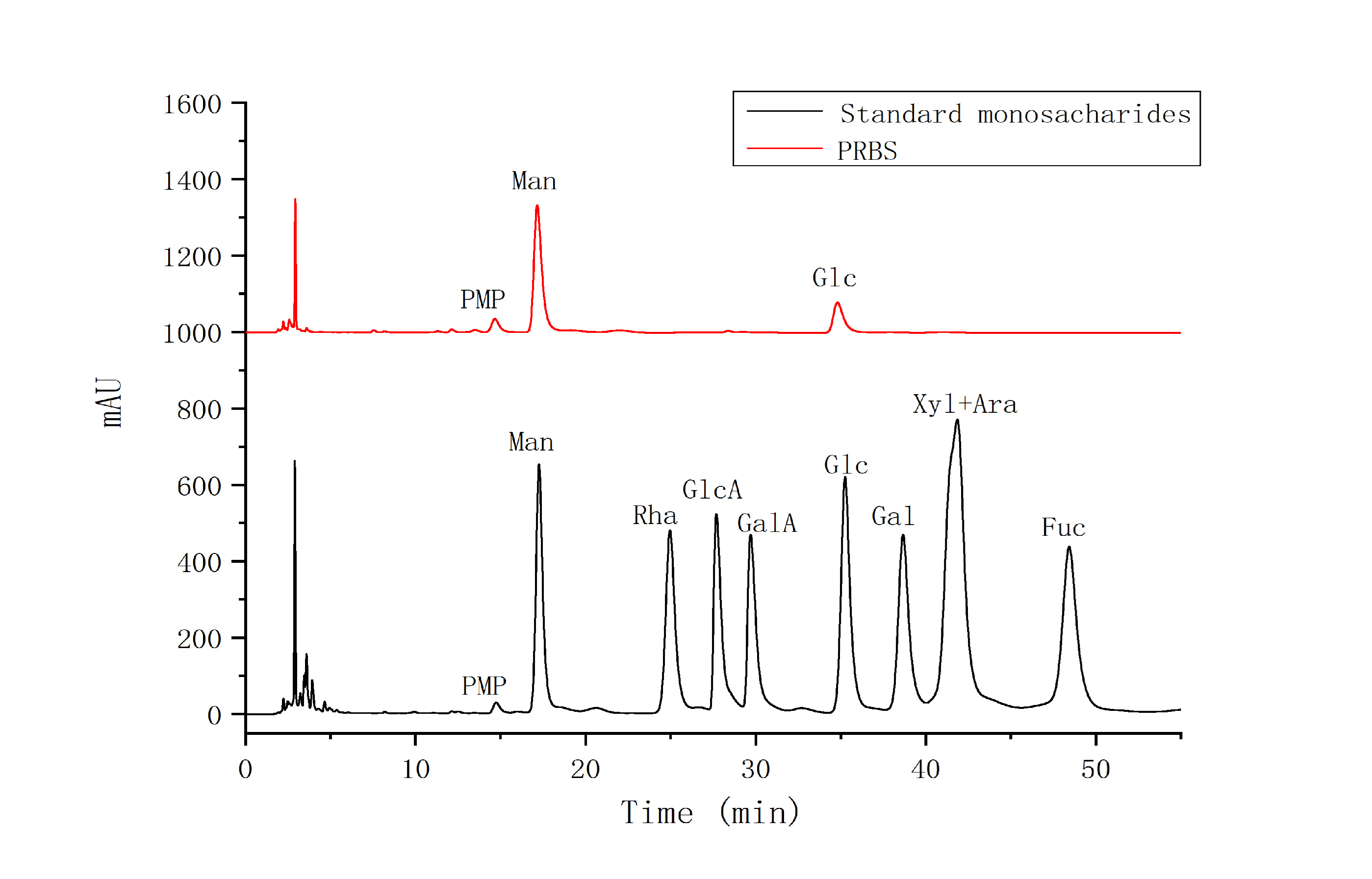
**

**Figure S4.** HPLC analysis of monosaccharide standards and PRBS after PMP derivation. Chromatographic conditions: sample: acid-hydrolysis PRBS 6 mg/ml; chromatographic column: Agilent-ZORBAX-SB-C18 4.6*250 mm; mobile phase: water 100% isocratic elution; Elution time: 30 min; Injection volume: 10 μL.


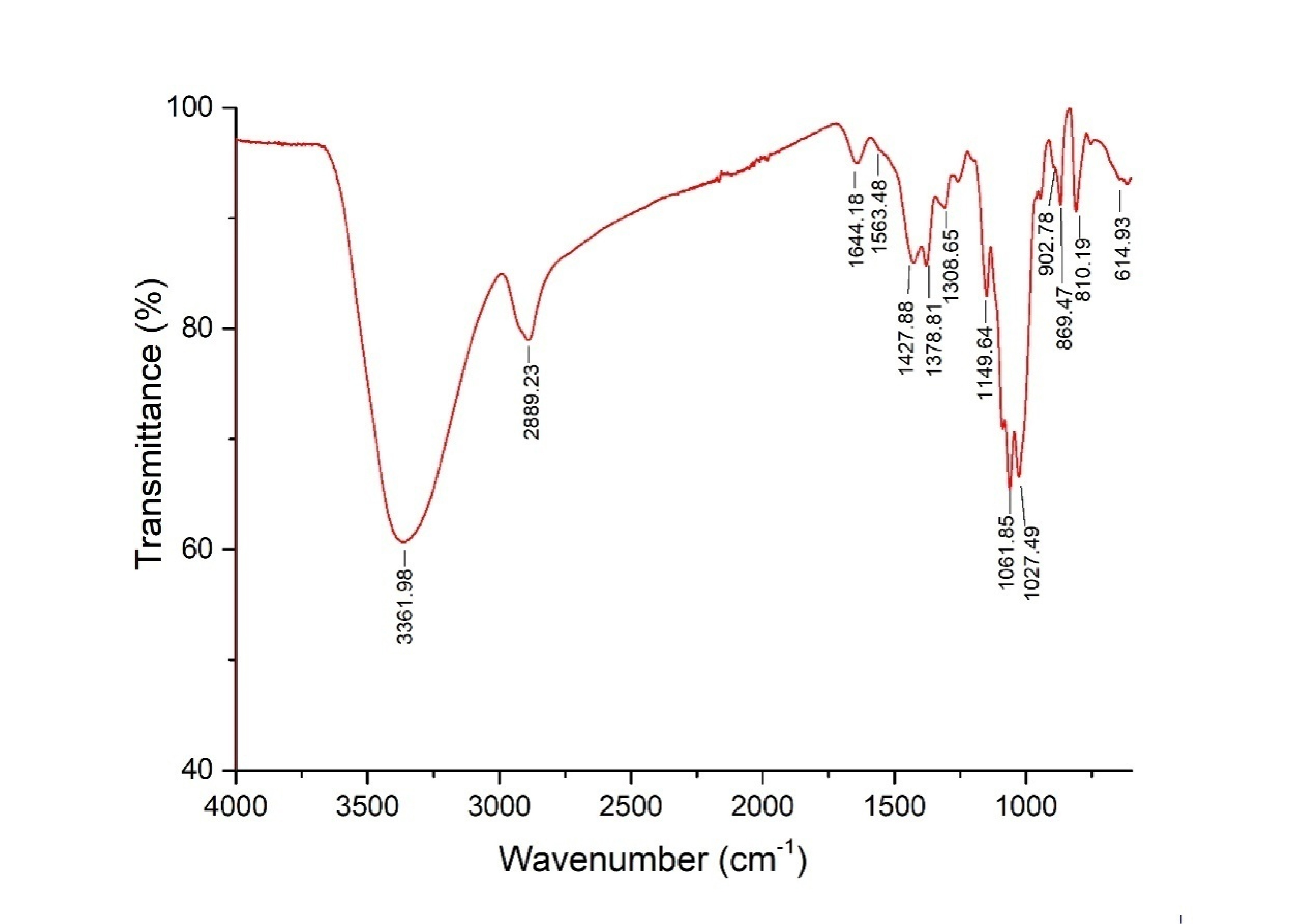


**Figure S5.** FT-IR spectra showing the characteristic absorption peaks of PRBS such as hydroxyl and carbony l.20 μL PRBS (2 mg/mL) aqueous solution mixed with KBr powder, dried and pressed, then detected by FT-IR.

Table S3. Chemical shifts for the resonances of glycosyl residues of PRBS ^1^H /^13^C NMR spectra.

| Sugar residues | →4)-β-D- glucose-(1→ | →4)-β-D-mannose-(1→ | →4)-β-D-mannose-(1→ |
| --- | --- | --- | --- |
| H-1 | 4.42(1H; CH) | 4.66(1H; CH) | 4.63(1H; CH) |
| C-1 | 102.48 | 100.10 | 99.94 |
| H-2 | 3.26(1H; CH) | 4.03(1H; CH) | 3.90/5.09(1H; CH) |
| C-2 | 72.72 | 69.89 | 70.45 |
| H-3 | 3.60(1H; CH) | 3.72(1H; CH) | 3.67(1H; CH) |
| C-3 | 73.85 | 71.40 | 71.44 |
| H-4 | 3.56(1H; CH) | 3.72(1H; CH) | 3.72(1H; CH) |
| C-4 | 78.44 | 76.51 | 76.41 |
| H-5 | 3.52(1H; CH) | 3.47(1H; CH) | 3.47(1H; CH) |
| C-5 | 74.64 | 74.98 | 75.18 |
| H-6 | 3.73(2H; CH2) | 3.82, 3.65(2H; CH2) | 3.92(2H; CH2) |
| C-6 | 60.04 | 60.47 | 60.25 |


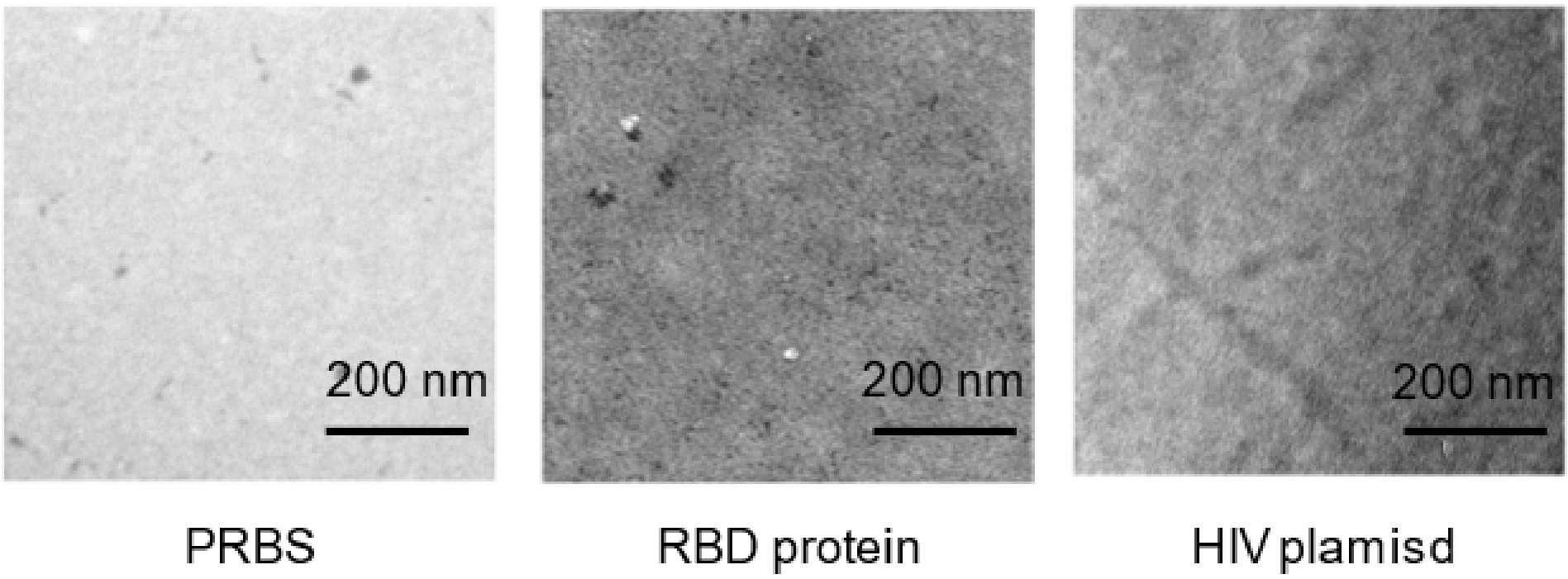


**Figure S6.** TEM images of naked PRBS, RBD protein and HIV plasmid.


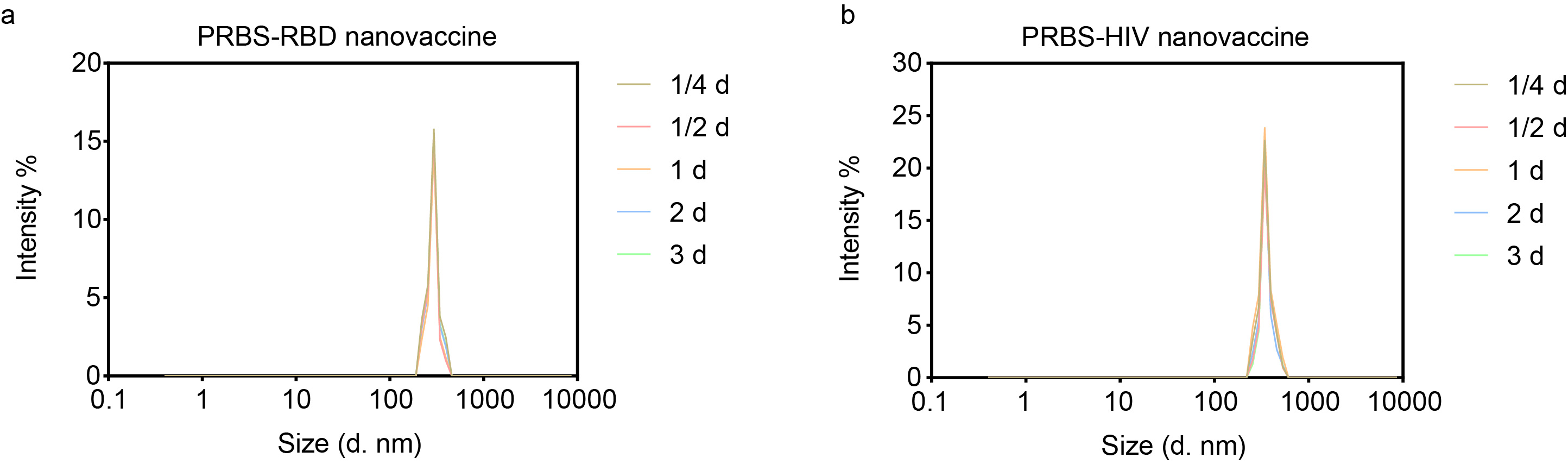


**Figure S7.** **(a)** DLS exhibited that the hydrate size of PRBS-RBD protein nanovaccine on 1/4,1/2,1,2,3 days. **(b)** DLS exhibited that the hydrate size of PRBS-HIV plasmid nanovaccine on 1/4,1/2,1,2,3 days.

Table S4. Hydrogen-bonds between PRBS and RBD

| RUN | | H Bond Residue | Distance (Å) |
| --- | --- | --- | --- |
| 1 | TYR-473 | | 1.8 |
| 2 | TYR-473 | | 2.2 |
| 3 | ARG-457 | | 2.6 |
| 4 | ARG-457 | | 1.8 |
| 5 | LEU-455 | | 2.0 |
| 6 | LYS-417 | | 2.3 |
| 7 | LYS-417 | | 2.4 |

**Interactions among PRBS and DNA**

**Figure S8.** Intermolecular interactions of the PRBS docked to HIV plasmid. Hydrophobic interactions between PRBS and HIV plasmid (deoxyadenine-6, deoxyguanine-22, and deoxycytosine-23) are mediated by water molecules. Intermolecular interactions are marked with dashed lines. The distances among heavy (non-hydrogen) atoms are quantified at the Angstroms level (Å).


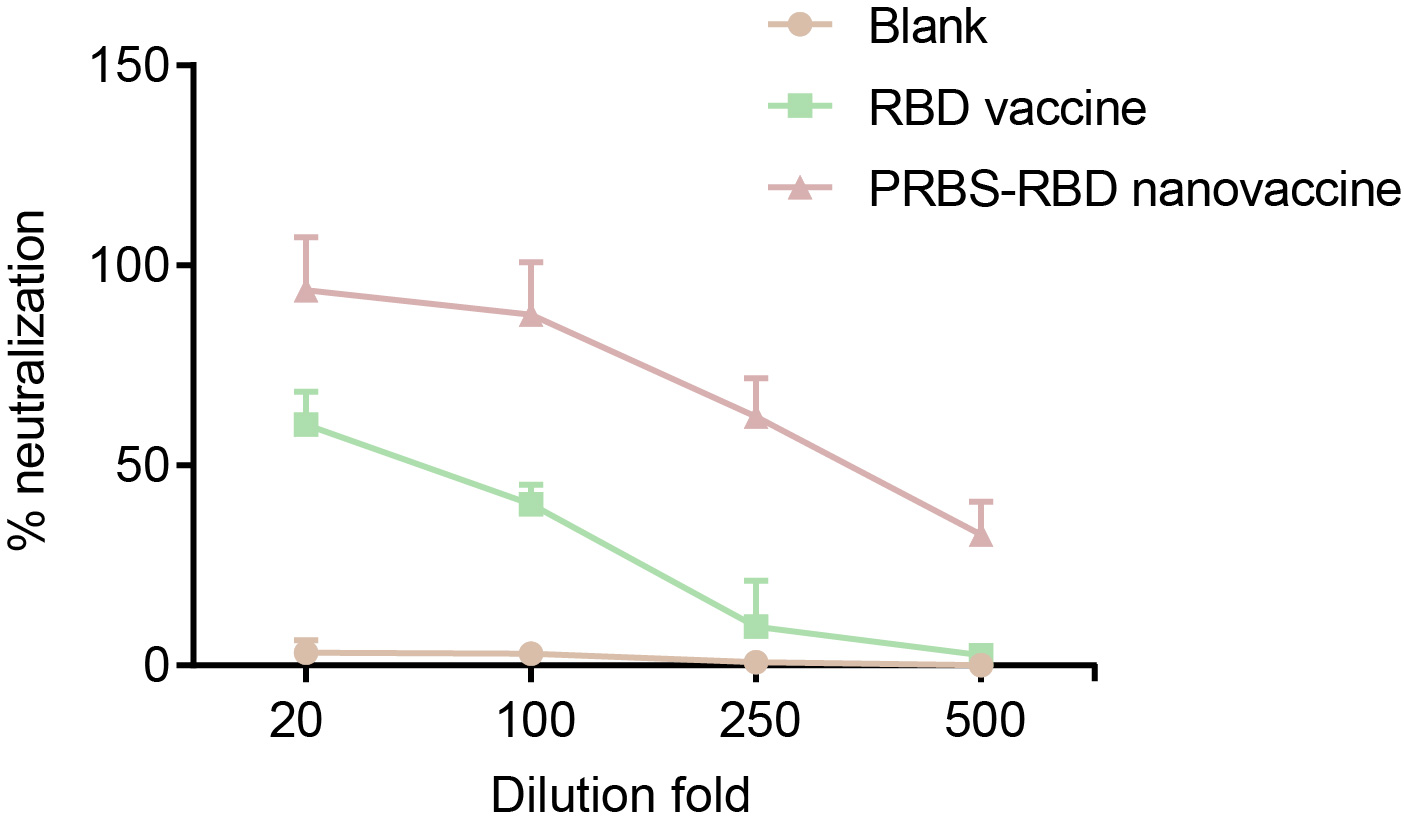


**Figure S9**. The inhibitory concentration curves of anti-SARS-CoV-2 neutralizating antibodies induced by either PRBS-RBD protein nanovaccine (10 μg RBD protein and 50 μg PRBS per each injection) or traditional RBD protein vaccine (10 μg RBD protein per each injection).


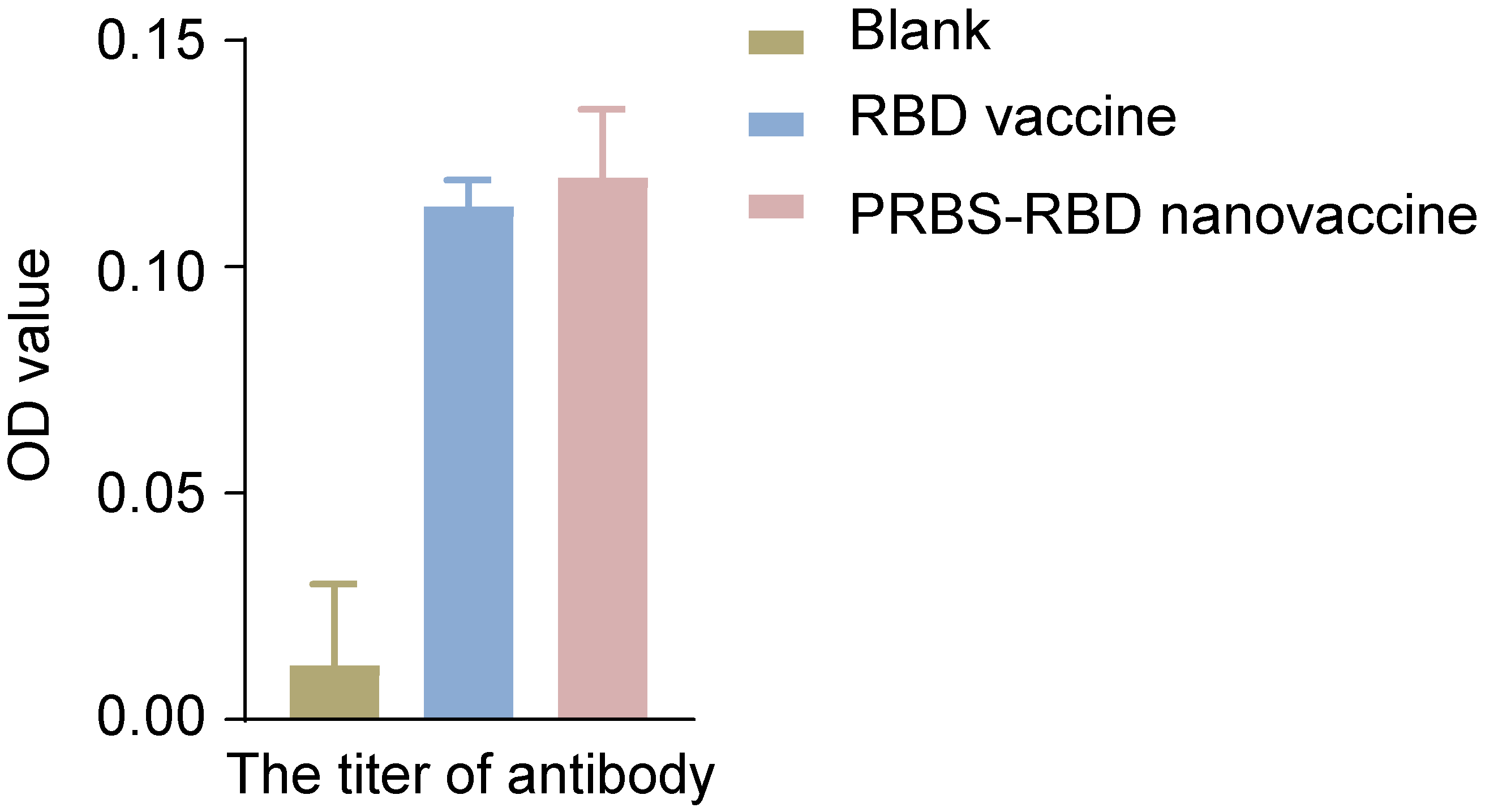


**Figure S10**. IgG responses in three mouse vaccination groups: Blank group (100 μL saline per each injection), RBD vaccine (10 μg RBD protein per each injection), and 100 μL PRBS-RBD nanovaccine (10 μg RBD protein and 50 μg PRBS). All groups’ data from 1:10000 dilution.


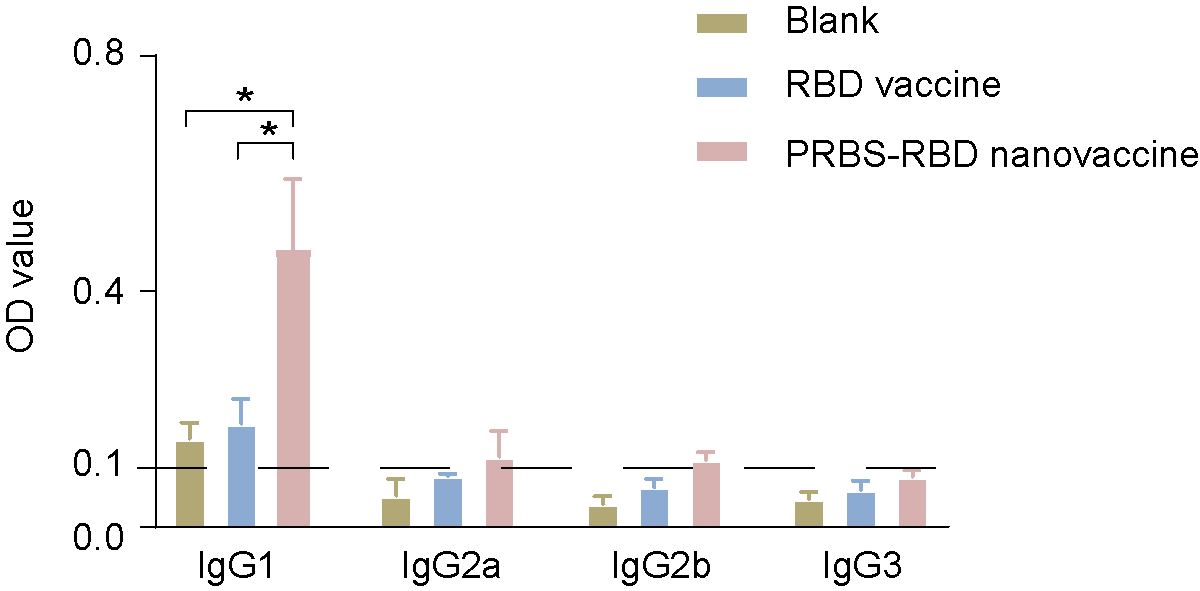


**Figure S11**. IgG subtype responses (IgG1, IgG2a, IgG2b, IgG3) in three mouse vaccination groups: Blank group (100 μL saline per each injection), RBD vaccine (10 μg RBD protein per each injection) and 100 μL PRBS-RBD nanovaccine (10 μg RBD protein and 50 μg PRBS). All groups’ data from 1:1000 dilution. All values are expressed as mean ± SEM for three duplicates. *P < 0.05 versus the control group.


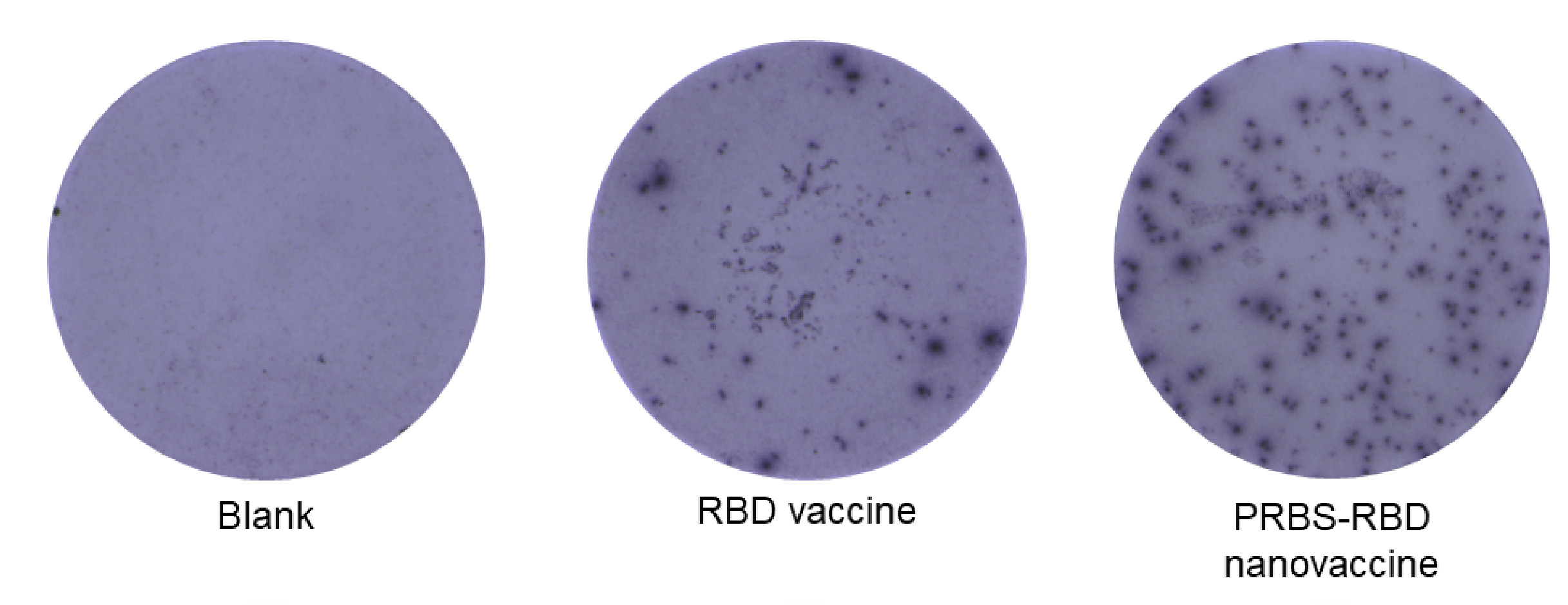


**Figure S12**. The specific IFN-γ ELISPOT photography results of RBD-specific T cell response induced by either PRBS-RBD protein nanovaccine (10 μg RBD protein and 50 μg PRBS per each injection) or traditional RBD protein vaccine (10 μg RBD protein per each injection).


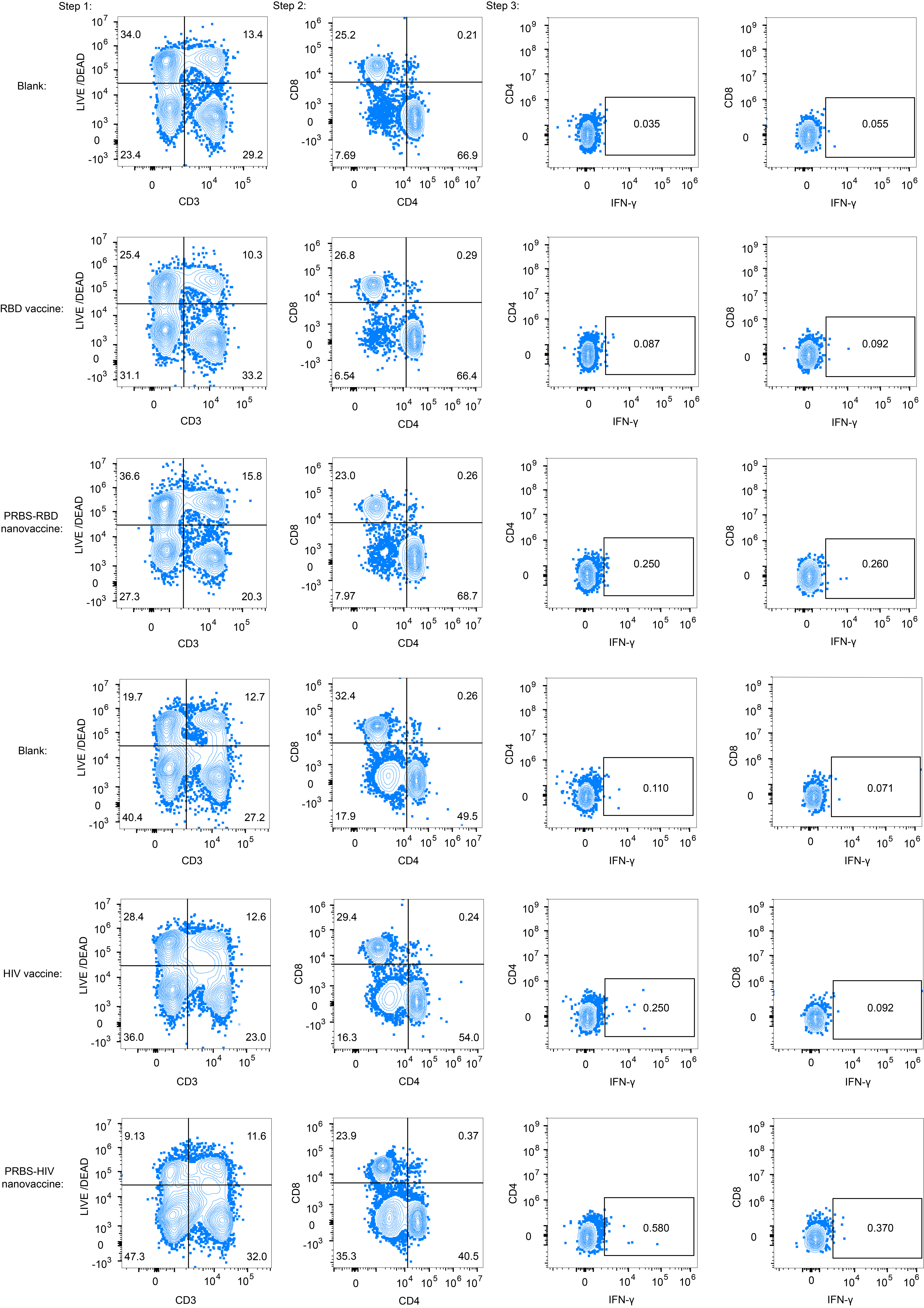


**Figure S13.** The representative gating strategy between parallel groups. Step 1: Live CD3^+^ T cells were selected on the basis of CD3^+^ and Zombie NIR^-^. Step 2: CD4 and CD8 were applied to define CD4 cells (CD4^+^CD8^-^) and CD8 cells (CD4^-^CD8^+^). Step 3: The percentages of CD4 and CD8 that expressed IFN-γ were displayed.


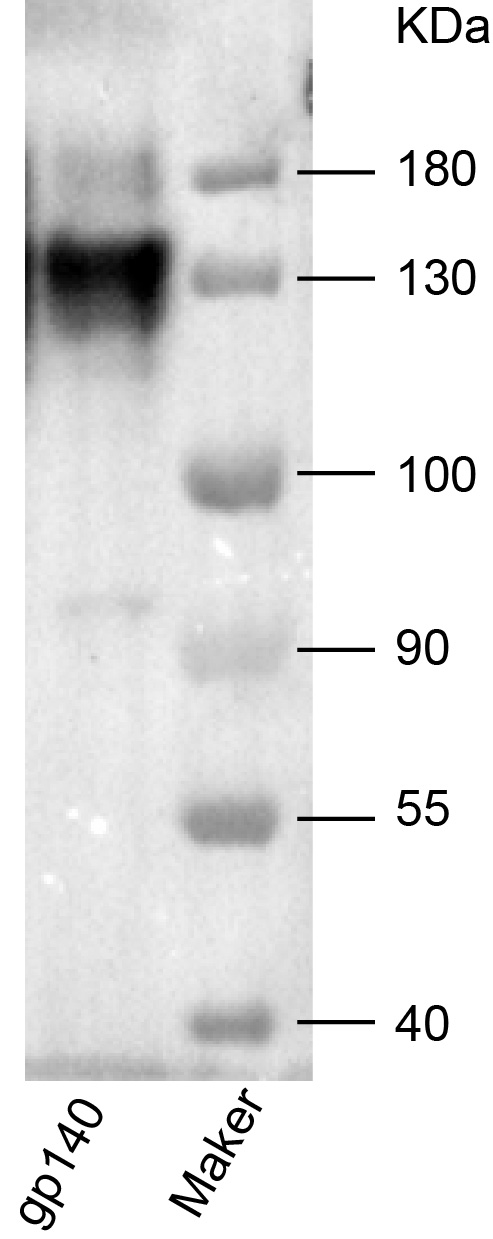


**Figure S14**. Western blotting results of HIV env plasmid expressing gp140 protein


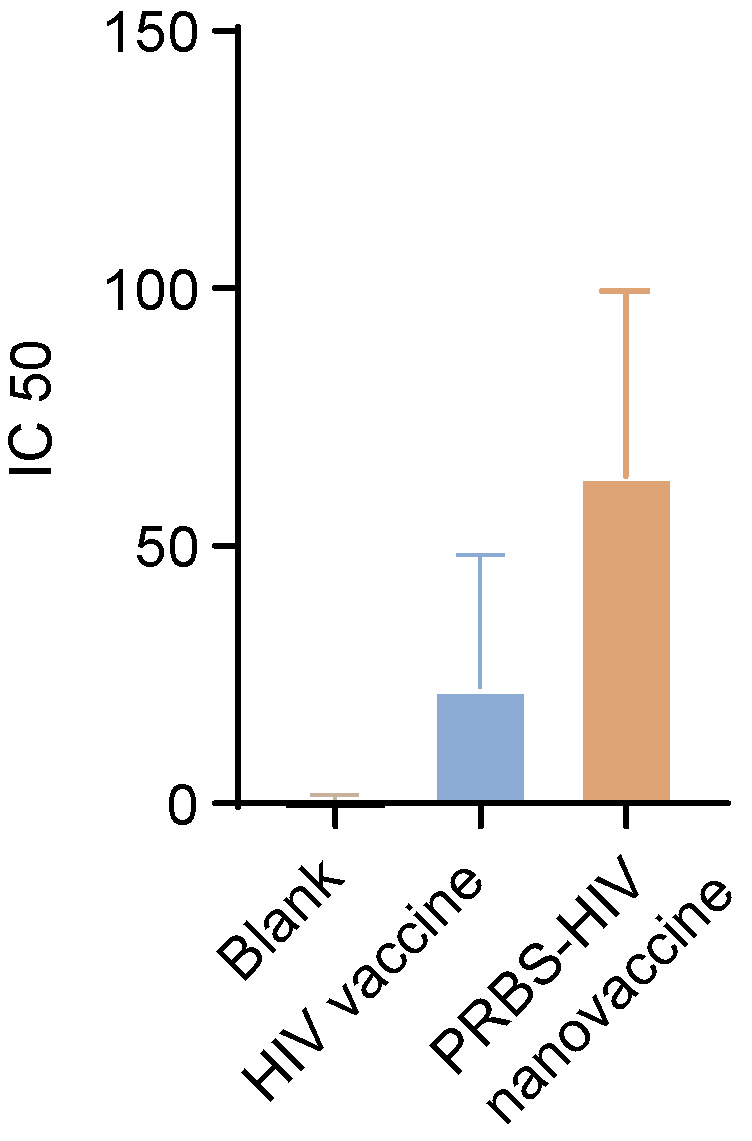


**Figure S15.** The IC_50_ of anti-HIV neutralizing antibodies induced by either PRBS-HIV plasmid nanovaccine (10 μg HIV plasmid and 50 μg PRBS per each injection) or traditional HIV DNA vaccine (10 μg HIV plasmid per each injection)


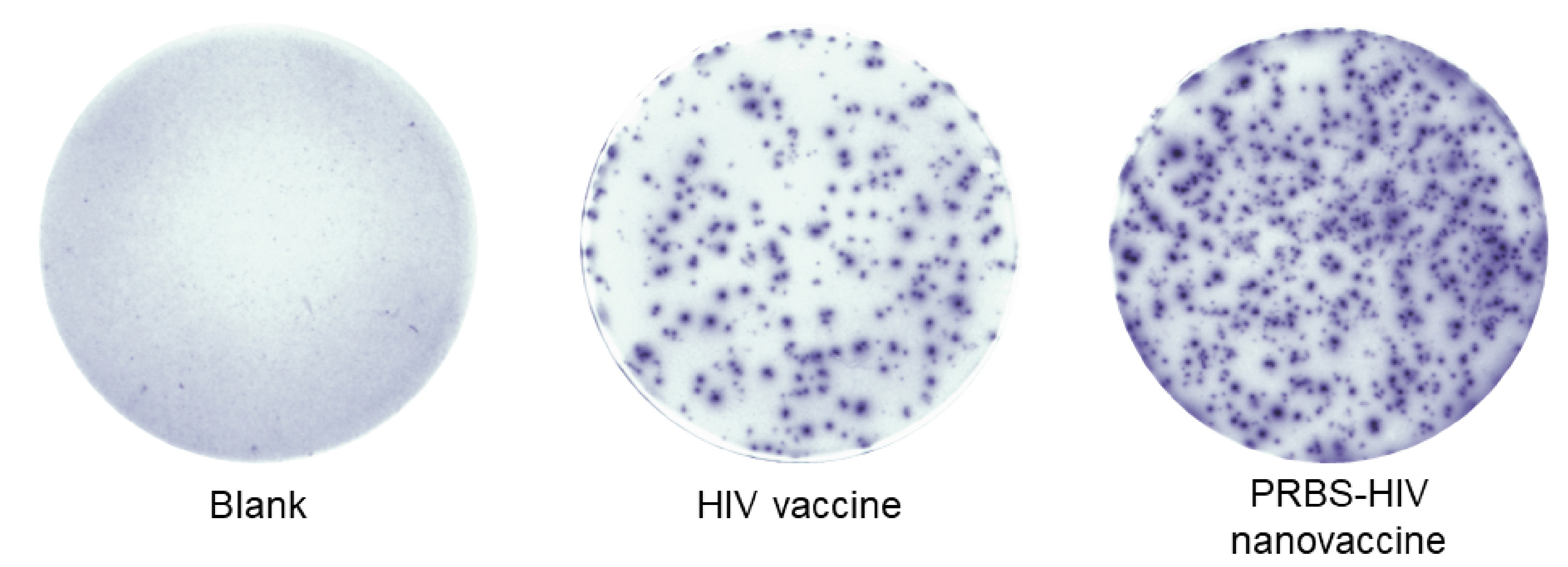


**Figure S16.** The specific IFN-γ ELISPOT photography results of HIV-specific T cell response induced by either PRBS-HIV plasmid nanovaccine (10 μg HIV plasmid and 50 μg PRBS per each injection) or traditional HIV DNA vaccine (10 μg HIV plasmid per each injection).


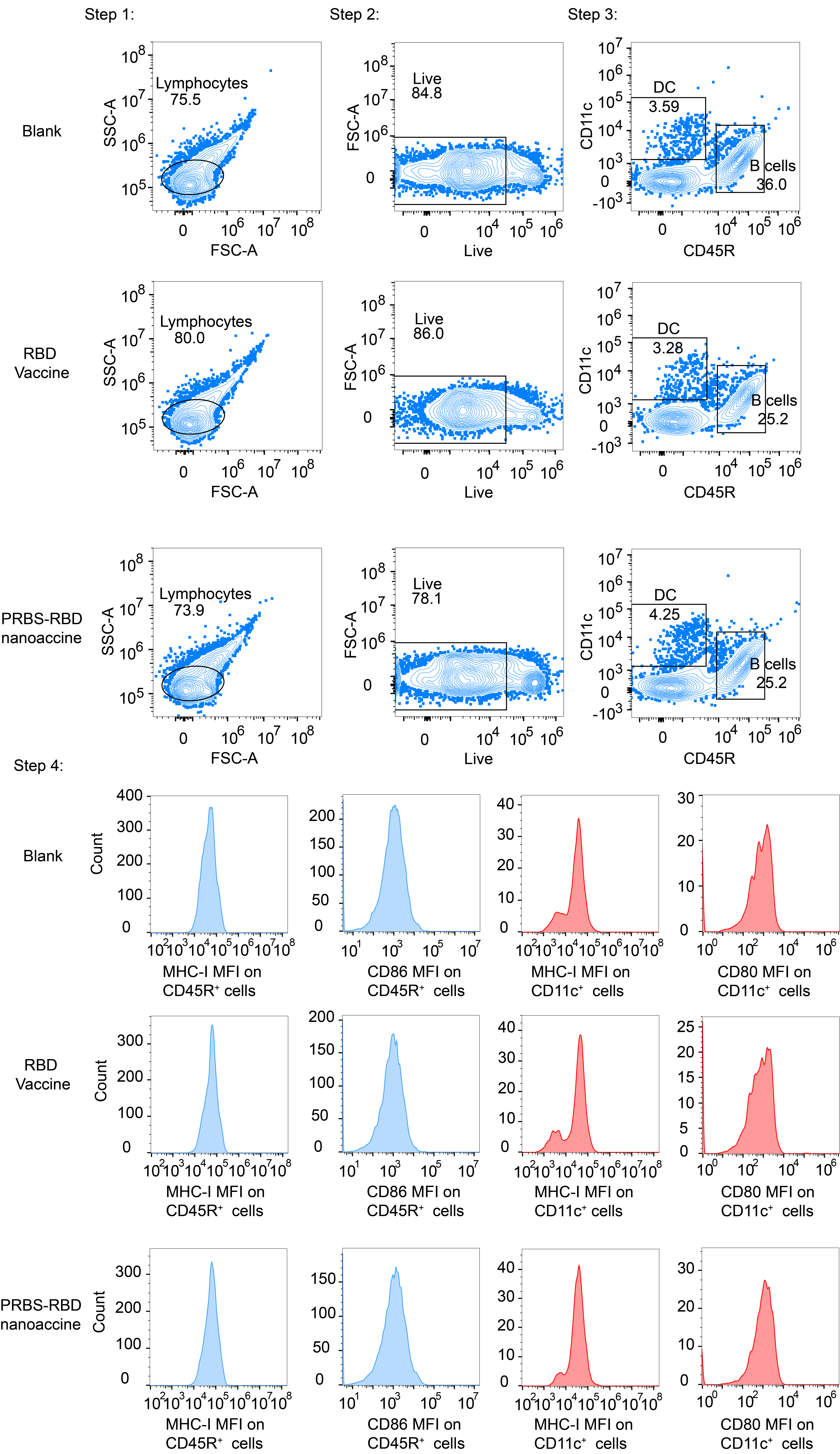


**Figure S17.** The representative detailed gating strategy and parallel groups results from either PRBS-RBD protein nanovaccine group (10 μg RBD protein and 50 μg PRBS per each injection) or traditional RBD protein vaccine group (10 μg RBD protein per each injection). Step 1: Lymphocytes were selected on the basis of FSC-A and SSC-A. Step 2: Dead cells were excluded by gating on Zombie NIR^+^. Step 3: CD11c and CD45R were applied to define DC cells (CD11c^+^CD45R^-^) and B cells (CD11c^-^CD45R^+^). Step 4: The mean fluorescent intensity (MFI) of MHC-I and CD86 on B cells and the mean fluorescent intensity (MFI) of MHC-I and CD80 on DCs were shown.


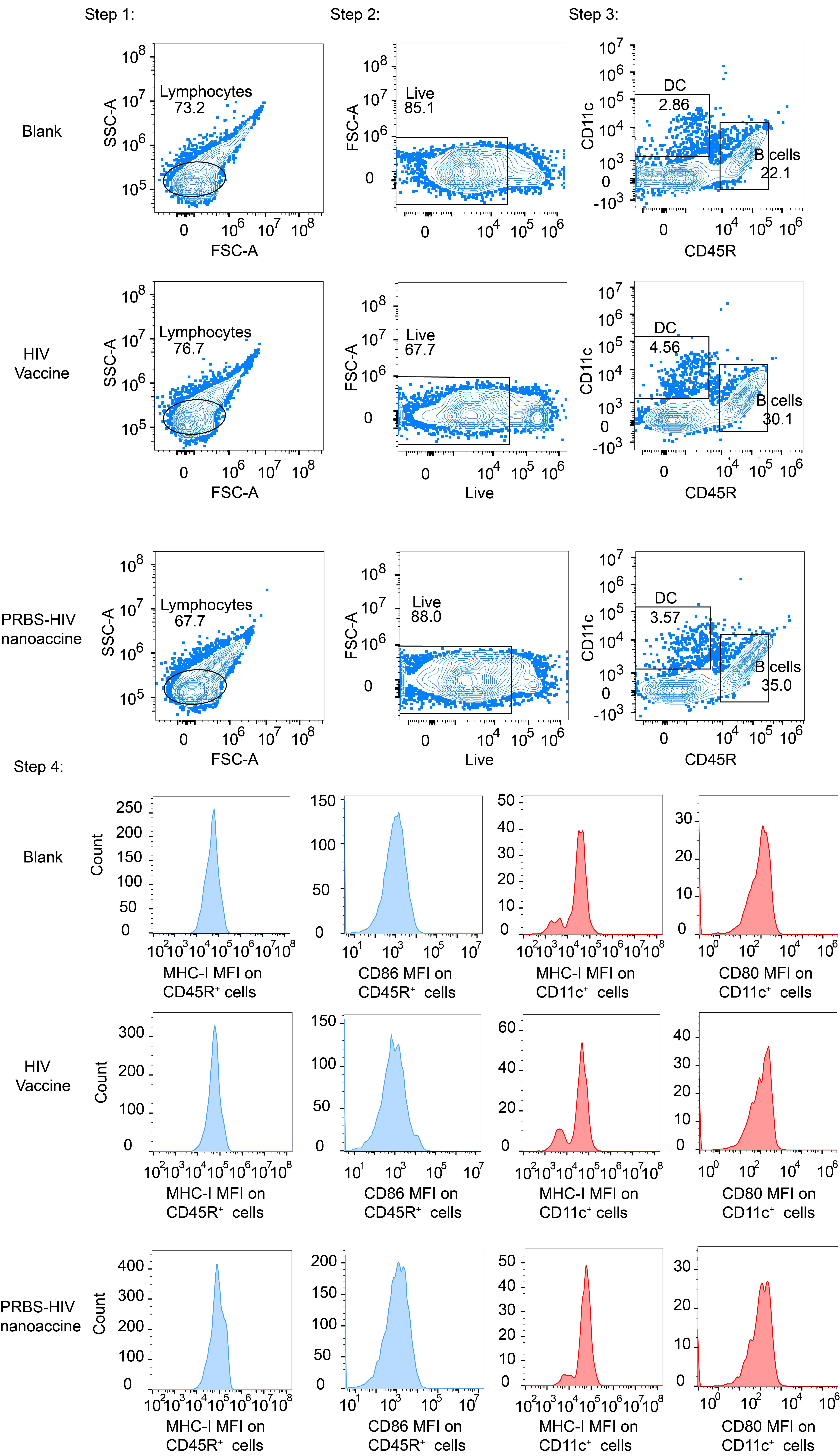


**Figure S18.** The representative detailed gating strategy and parallel groups results from either PRBS-HIV plasmid nanovaccine group (10 μg HIV plasmid and 50 μg PRBS per each injection) or traditional HIV plasmid vaccine group (10 μg HIV plasmid per each injection). Step 1: Lymphocytes were selected on the basis of FSC-A and SSC-A. Step 2: Dead cells were excluded by gating on Zombie NIR^+^. Step 3: CD11c and CD45R were applied to define DC cells (CD11c^+^CD45R^-^) and B cells (CD11c^-^CD45R^+^). Step 4: The mean fluorescent intensity (MFI) of MHC-I and CD86 on B cells and the mean fluorescent intensity (MFI) of MHC-I and CD80 on DCs were shown.


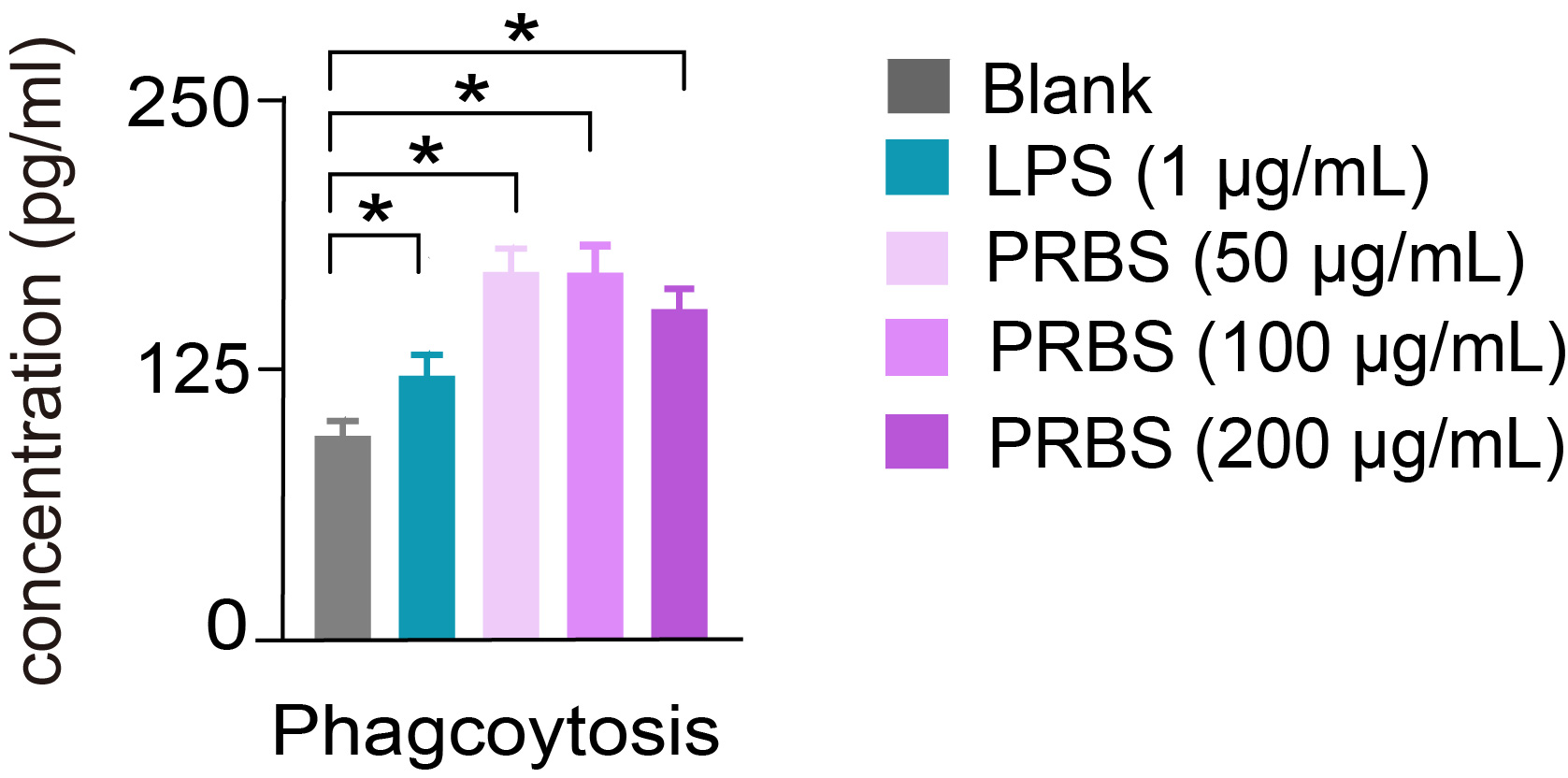


**Figure S19.** Different concentrations of PRBS (100 μg/mL or 200 μg/mL) promote the phagocytosis ability of macrophages. Saline as a negative control, LPS (1 μg/mL) was used as the positive control. All values are expressed as mean ± SEM for three duplicates. *P < 0.05 versus the control group.


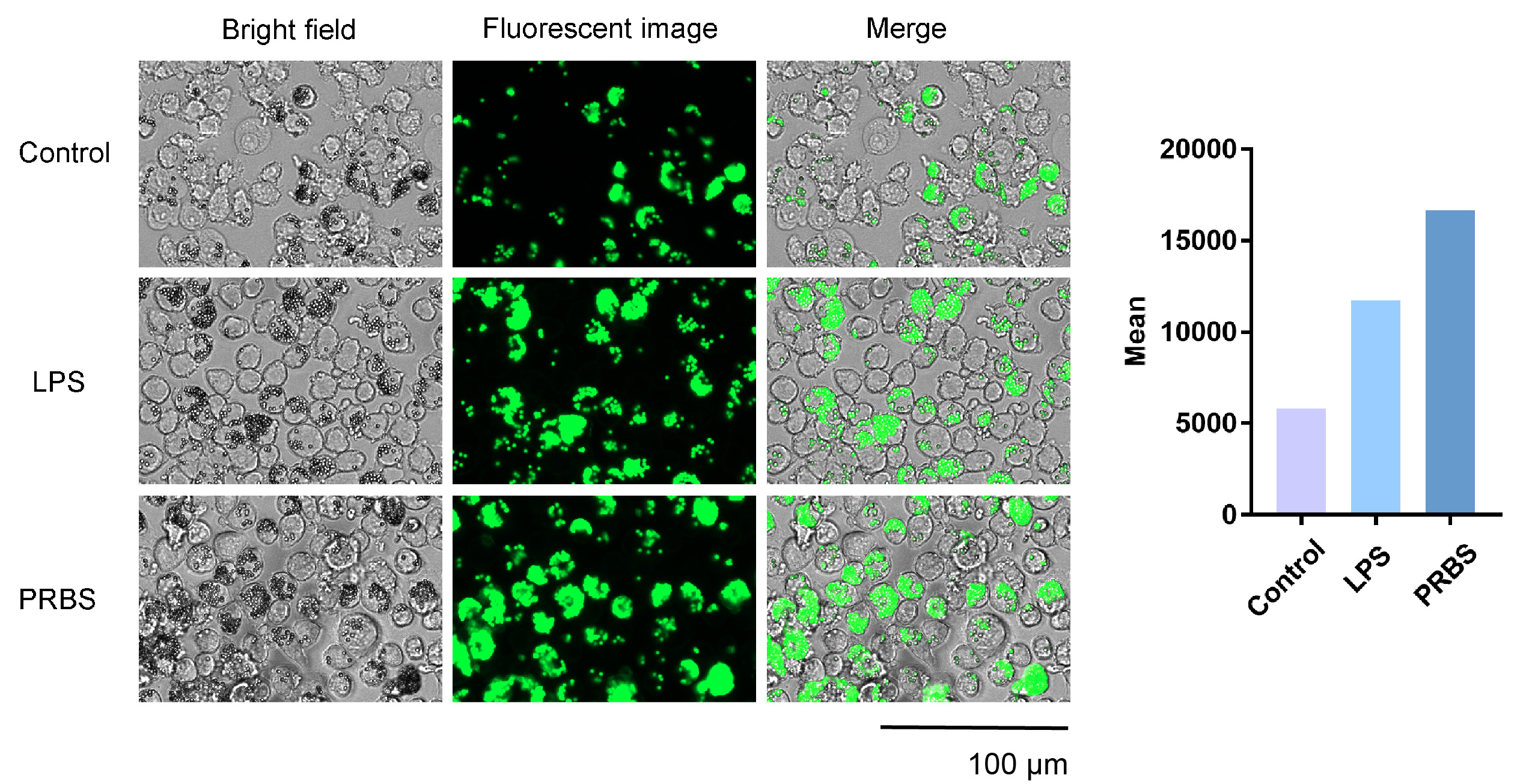


**Figure S20.** Fluorescence images for the phagocytosis of dendritic cells (DC2.4). Saline and 1 μg/mL LPS (a positive stimulator) were used as negative control and positive control. The value of fluorescence mean intensity from test samples.


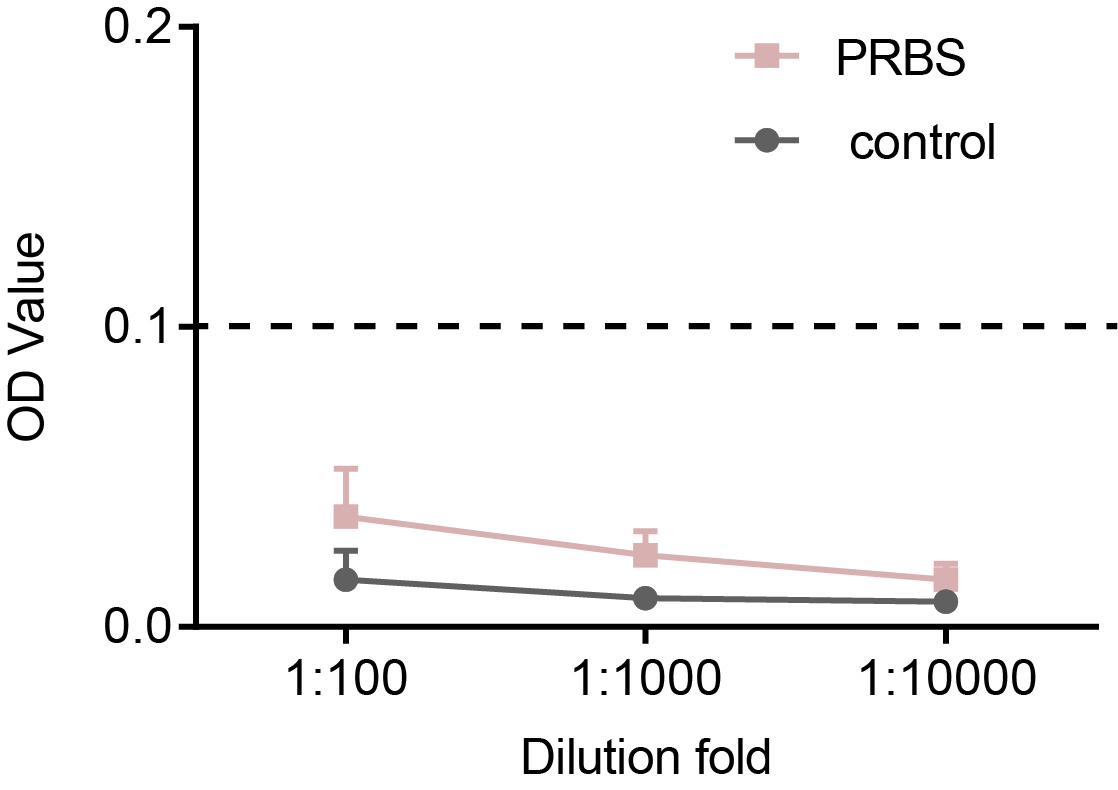


**Figure S21.** PRBS-specific IgG responses in two mouse vaccination groups: PRBS (50 μg PRBS per each injection), and negative control (100 μL salin per each injection).

**
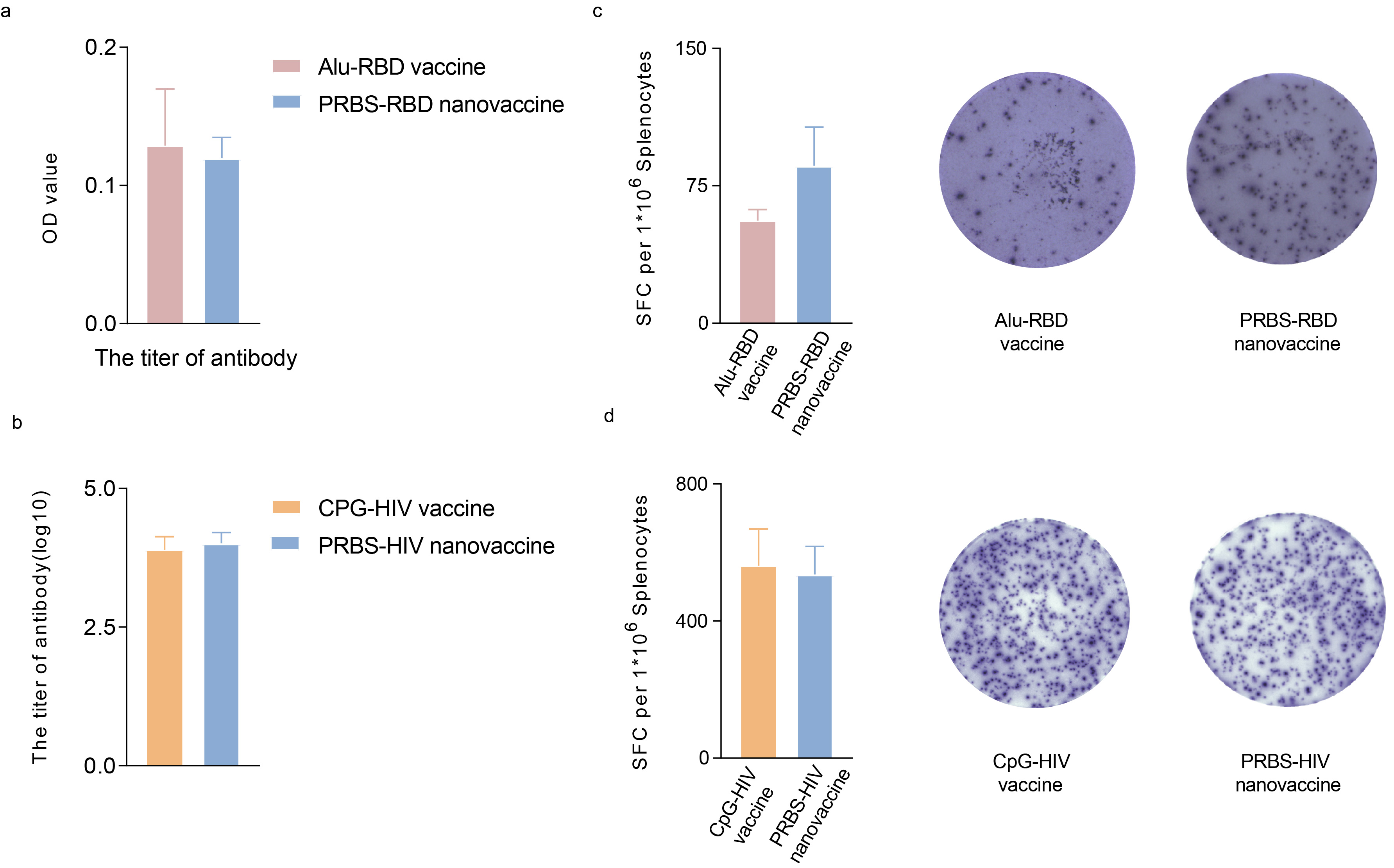
**

**Figure S22.** **(a)** IgG responses in two mouse vaccination groups: Alu-RBD vaccine (10 μg RBD protein and 35 μg Alu per each injection), and 100 μL PRBS-RBD nanovaccine (10 μg RBD protein and 50 μg PRBS per each injection). All groups’ data from 1:10000 dilution. **(b)** The titer of HIV-specific IgG induced by either CpG-HIV vaccine (15 μg CpG and 10 μg HIV plasmid per each injection) or 100 μL PRBS-HIV nanovaccine (10 μg HIV plasmid and 50 μg PRBS per each injection) **(c)** The magnitude of RBD-specific T cell response (IFN-γ ELISPOT) induced by either Alu-RBD vaccine (10 μg RBD protein and 35 μg Alu per each injection) or 100 μL PRBS-RBD nanovaccine (10 μg RBD protein and 50 μg PRBS per each injection). **(d)** The magnitude of HIV-specific T cell response (IFN-γ ELISPOT) induced by either CpG-HIV vaccine (15 μg CpG and 10 μg HIV plasmid per each injection) or 100 μL PRBS-HIV nanovaccine (10 μg HIV plasmid and 50 μg PRBS per each injection).
